# LARP7 enhances the potential of dental pulp stem cells to promote peripheral nerve repair

**DOI:** 10.1093/stmcls/sxag013

**Published:** 2026-03-11

**Authors:** Zihan Yang, Guanlin Qu, Xiping Wang, Li Wang, Lu Chen, Guiqiang Fu, Wenze Chen, Zitong Yang, Wenjing Li, Yuqiong Zhou, Jiacheng Jin, Linxi Zhou, Duohong Zou

**Affiliations:** Department of Oral Surgery, Shanghai Ninth People’s Hospital, Shanghai Jiao Tong University School of Medicine, College of Stomatology, Shanghai Jiao Tong University, National Center for Stomatology, National Clinical Research Center for Oral Diseases; Shanghai Key Laboratory of Stomatology, Shanghai, 200001, China; Department of Oral Surgery, Shanghai Ninth People’s Hospital, Shanghai Jiao Tong University School of Medicine, College of Stomatology, Shanghai Jiao Tong University, National Center for Stomatology, National Clinical Research Center for Oral Diseases; Shanghai Key Laboratory of Stomatology, Shanghai, 200001, China; Institute of Stomatology, School and Hospital of Stomatology, Wenzhou Medical University, Wenzhou, 325027, China; Department of Oral Surgery, Shanghai Ninth People’s Hospital, Shanghai Jiao Tong University School of Medicine, College of Stomatology, Shanghai Jiao Tong University, National Center for Stomatology, National Clinical Research Center for Oral Diseases; Shanghai Key Laboratory of Stomatology, Shanghai, 200001, China; Institute of Stomatology, School and Hospital of Stomatology, Wenzhou Medical University, Wenzhou, 325027, China; Department of Oral Surgery, Shanghai Ninth People’s Hospital, Shanghai Jiao Tong University School of Medicine, College of Stomatology, Shanghai Jiao Tong University, National Center for Stomatology, National Clinical Research Center for Oral Diseases; Shanghai Key Laboratory of Stomatology, Shanghai, 200001, China; Stomatology Hospital and College, Key Laboratory of Oral Diseases Research of Anhui Province, Anhui Medical University, Hefei, 230000, China; Stomatology Hospital and College, Key Laboratory of Oral Diseases Research of Anhui Province, Anhui Medical University, Hefei, 230000, China; Zhejiang Chinese Medical University School of Medical Technology and Information Engineering, Hangzhou, 310053, China; Department of Oral Surgery, Shanghai Ninth People’s Hospital, Shanghai Jiao Tong University School of Medicine, College of Stomatology, Shanghai Jiao Tong University, National Center for Stomatology, National Clinical Research Center for Oral Diseases; Shanghai Key Laboratory of Stomatology, Shanghai, 200001, China; Department of Oral Surgery, Shanghai Ninth People’s Hospital, Shanghai Jiao Tong University School of Medicine, College of Stomatology, Shanghai Jiao Tong University, National Center for Stomatology, National Clinical Research Center for Oral Diseases; Shanghai Key Laboratory of Stomatology, Shanghai, 200001, China; Touro College of Dental Medicine at New York Medical College, Hawthorne, NY 10532, USA; Department of Orthodontics, Shanghai Ninth People’s Hospital, Shanghai Jiao Tong University School of Medicine, College of Stomatology, Shanghai Jiao Tong University, National Center for Stomatology, National Clinical Research Center for Oral Diseases, Shanghai Key Laboratory of Stomatology, Shanghai, 200001, China; Department of Oral Surgery, Shanghai Ninth People’s Hospital, Shanghai Jiao Tong University School of Medicine, College of Stomatology, Shanghai Jiao Tong University, National Center for Stomatology, National Clinical Research Center for Oral Diseases; Shanghai Key Laboratory of Stomatology, Shanghai, 200001, China

**Keywords:** stem cell therapy, mesenchymal stem cell, tissue regeneration, regenerative medicine, peripheral nerve injuries

## Abstract

**Background:**

Peripheral nerve injuries (PNIs) present a persistent clinical challenge due to the intrinsically limited regenerative capacity of peripheral nerves. While dental pulp stem cells (DPSCs) exhibit significant neuroregenerative potential, their therapeutic efficacy is constrained by hostile microenvironments and inherent functional heterogeneity. Genetic modification may offer a promising strategy to enhance their therapeutic capabilities.

**Methods:**

DPSCs were induced toward neural lineage differentiation, and key gene candidates were identified through qRT-PCR. Lentiviral-mediated gene interference was performed to modulate target gene expression, followed by comprehensive analysis of differentiation outcomes using qRT-PCR, Western blotting, and immunofluorescence assays. RNA sequencing was employed to uncover associated signaling pathways, which were subsequently validated through pharmacological inhibition with specific inhibitors. The therapeutic efficacy of genetically engineered DPSCs was evaluated in a rat model of sciatic nerve crush injury, with neural regeneration quantitatively assessed via neuroelectrophysiological measurements and histological analyses.

**Results:**

LARP7 positively regulated the Schwann cell-like differentiation of DPSCs, as well as their trophic and anti-inflammatory effects, thus enhancing its therapeutic effects on nerve repair and promoting functional recovery. Mechanistically, we found that LARP7 remodeled cytokine-cytokine receptor interactions, enhancing trophic support while attenuating proinflammatory responses, and activated the PI3K-Akt-mTOR signaling pathway, with ERBB4 serving as a critical downstream effector, promoting DPSCs differentiation into Schwann cell-like phenotypes.

**Conclusions:**

Collectively, LARP7-mediated changes in DPSCs establish a new therapeutic paradigm that addresses the limitations of current stem cell-based interventions and enables the development of standardized biotherapeutics for peripheral nerve repair.

## Graphical Abstract

**Graphical Abstract:**
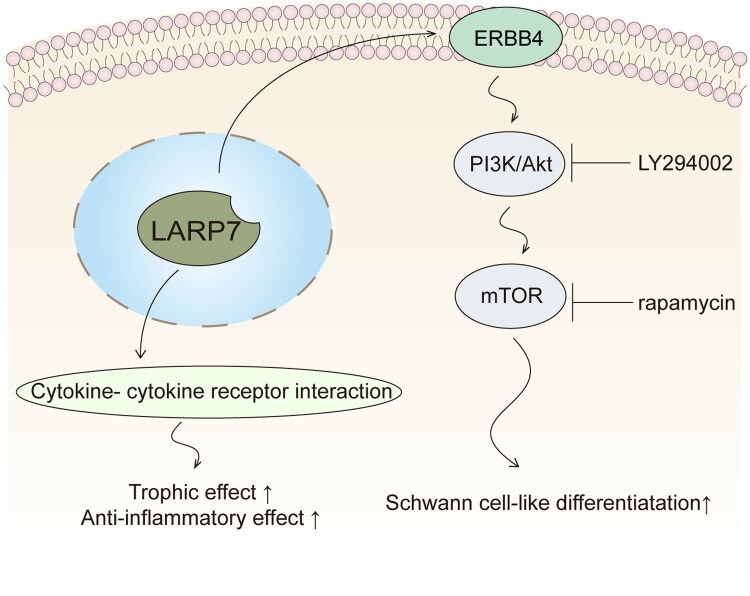

Significance statement Peripheral nerve repair remains constrained by Schwann cell phenotypic atrophy and regenerative microenvironmental imbalances. This study demonstrates that LARP7 critically enhances dental pulp stem cell (DPSC)-based therapy through dual mechanisms: (1) Driving differentiation into functional Schwann-like phenotypes to replenish neural deficits; and (2) Amplifying trophic support and anti-inflammatory modulation to synergistically remodel the injury niche. These findings innovatively address two major hurdles in nerve regeneration—cellular replacement and microenvironmental dysregulation—via coordinated molecular intervention. The LARP7-mediated DPSC-based strategy elevates axonal regrowth efficiency and locomotor recovery while resolving transplant survival and heterogeneity challenges. This work provides a template for developing standardized regenerative protocols and accelerates the clinical translation of tissue-engineered nerve grafts.

## Introduction

Peripheral nerve injuries (PNIs) result in persistent sensory and motor disabilities, representing a significant clinical challenge worldwide with profound patient morbidity and socioeconomic burden.^1–3^ Current management strategies rely primarily on pharmacological and surgical interventions^4^^,^^5^ but often fail to achieve optimal functional recovery.^6^^,^^7^ These findings underscore the critical need for innovative nerve repair approaches.

Schwann cells serve as essential functional units in peripheral nerves, sustaining axonal integrity under physiological conditions and orchestrating post-injury regeneration.^8^^,^^9^ However, chronic denervation triggers Schwann cell dysfunction and phenotypic atrophy.^10^ While Schwann cell transplantation has regenerative potential,^11–13^ clinical translation is constrained by donor scarcity and limited proliferative capacity.^14^

Mesenchymal stem cells have emerged as promising candidates for peripheral nerve regeneration,^15–17^ with proposed mechanisms including cell replacement, trophic factor secretion, remyelination, microenvironment modulation, and immunoregulatory functions.^10^^,^^18^ Dental pulp stem cells (DPSCs), derived from neural crest-origin dental pulp, exhibit exceptional self-renewal, multilineage differentiation, injury-site homing, and immunomodulatory properties through paracrine activity.^19^ Crucially, DPSCs share an embryonic lineage with peripheral glia and functionally replenish Schwann cells within the dense neural network of the dental pulp,^20–22^ suggesting inherent neuroregenerative competence. Although DPSCs demonstrate neural differentiation potential and partial repair capability,^23^ their therapeutic efficacy in PNIs remains limited by hostile microenvironments and inherent cellular heterogeneity.^24^^,^^25^

Current regenerative strategies focus on the genetic enhancement of stem cells to augment therapeutic potency.^26^ Among potential molecular targets, La ribonucleoprotein 7 (LARP7) is expressed predominantly in the developing central nervous system, with deficiency causing embryonic neurodegeneration,^27^ and is upregulated during early neuronal differentiation of embryonic stem cells.^28^ Despite these neural associations, the role of LARP7 in modulating DPSCs for nerve repair remains unexplored.

In this study, we identified LARP7 as a positive regulator that enhances the nerve repair capacity of DPSCs. Mechanistically, LARP7 activates the PI3K-Akt-mTOR signaling pathway via ERBB4 and modulates cytokine-cytokine receptor interactions, thereby promoting Schwann cell-like differentiation, increasing trophic support, and attenuating pro-inflammatory responses. These findings highlight LARP7 as a potential therapeutic target to overcome current limitations in DPSC-based strategies for peripheral nerve repair.

## Methods

### Isolation and culture of DPSCs

Third molars from healthy adults aged 18-25 were collected post-therapy. The study was approved by the Research Ethics Committee of Shanghai Jiao Tong University School of Medicine (SH9H-2019-T167-6). After surface cleaning, pulp tissue was extracted from the opened pulp chamber, minced, and digested with 3 mg/mL collagenase type I (Biofroxx, Germany) in Hanks’ solution for 40 min at 37 °C. Digestion was stopped by centrifugation (1000 rpm, 5 min), and the pellet was resuspended in α-MEM supplemented with 20% FBS and 100 U/mL penicillin-streptomycin (Beyotime, China). Cells were plated in 100-mm dishes and cultured at 37 °C/5% CO_2_. At 80%-90% confluence, they were passaged 1:3 using 0.25% trypsin in α-MEM + 10% FBS. For storage, cells were frozen in serum-free cryopreservation medium (NCM C40100) at −80 °C and used between passages 3 and 5.

### Neural differentiation

DPSCs (1 × 10^5^ cells/well) were seeded in 6-well plates. After 24 h, the 10% FBS MEM-α medium was replaced with neural differentiation medium: Neurobasal medium (21103049, Gibco, USA) supplemented with 2% B27 (A3582801, Gibco, USA), 20 ng/mL bFGF (13256-029, Gibco, USA), and 20 ng/mL EGF (PHG0311, Gibco, USA). The medium was changed every 3 days. DPSCs were treated with LY294002 (25 μM; Selleckchem, S1105), rapamycin (200 nM; Selleckchem, S1039), AZ-5104 (10 nM, MedChemExpress, HY-B0793), EF-1 (10 μM, MedChemExpress, HY-168438), or DMSO for 72 h. Cells were harvested for protein and RNA extraction.

### Generation of stable LARP7 overexpressing/knockdown DPSCs

Lenti-OE-LARP7 and Lenti-sh-LARP7 as well as control vectors were constructed and purchased from Hanbio (Shanghai, China). sh-LARP7-upper chain: 5'-gatccGAGCTAGCATGGCTTCTTTAAActcgagTTTAAAGAAGAAGCCATGCTAGCTttttg-3'; sh-LARP7-lower chain: 5'-aattcaaaaaaAGCTAGCATGGCTTCTTTAAActcgagTTAAAGAAGCCATGCTAGAGCg-3'. Stable transfection was performed following the manufacturer’s instructions. Briefly, lentivirus and polybrene (2 μg/mL, Hanbio) were added to the culture medium and incubated with DPSCs at a multiplicity of infection of 5 for 48 h. Stably transfected cells were then screened with puromycin for 3 days.

### Quantitative RT-PCR (qRT-PCR)

Total RNA was extracted from the samples via RNAiso (Takara, Japan) and subsequently converted into cDNA with a reverse transcription kit (Yisheng, China). qRT-PCR was performed on a Light-Cycler 480 PCR System (Roche, CH) using SYBR Green I master mix (Yisheng, China). *ACTB* was used to normalize expression. The primers utilized in the qRT-PCR assays are listed in Table S1.

### Western blot (WB)

Protein lysates were extracted from DPSCs with RIPA buffer containing protease and phosphatase inhibitors, and a Bradford protein assay kit (Sangon Biotech, China) was used to determine the total protein concentration. Equal quantities of proteins were subjected to electrophoresis on 10%, 12.5%, or 15% polyacrylamide gels (EpiZyme Biotechnology, China), transferred onto a nitrocellulose membrane, and incubated with antibodies against GAPDH (Rabbit, 1:5000, HX1832, HuaXingbio), NES (Mouse, 1:1000, ab22035, Abcam), PAX6 (Rabbit, 1:2000, 12323-1-AP, Proteintech), TUBB3 (Rabbit, 1:5000, ab133582, Abcam), GAP43 (Rabbbit, 1:2000, DF7766, Affinity), SOX10 (Mouse, 1:2000, 66786-1-lg, Proteintech), NGFR (Rabbit, 1:1000, #8238S, CST), GFAP (Rabbit, 1:500, ab207165, Abcam), S100B (Mouse, 1:1000, S2532, Sigma-Aldrich), BDNF (Rabbit, 1:2000, A4873, ABclonal), LARP7 (Rabbit, 1:2000, 17067-1-AP, Proteintech), p-PI3K(Rabbit, 1:2000, T40116, Abmart), PI3K(Rabbit, 1:2000, T40115, Abmart), p-Akt (Rabbit, 1:1000, AF887, R&D system), Akt (Mouse, 1:1000, MB2055, R&D system), p-mTOR (Rabbit, 1:2000, 5536, CST), and mTOR (Rabbit, 1:2000, 2983, CST) at 4 °C overnight. The membranes were subsequently incubated with HRP-conjugated anti-rabbit IgG (1:4000, HX2031, HuaXingbio) or anti-mouse IgG (1:5000, HX2032, HuaXingbio) secondary antibodies for 1 h at RT. Immunoreactive bands were detected via enhanced chemiluminescence (ECL, Biosharp) with the ChemiDoc imaging system (Bio-Rad, USA).

### Immunofluorescence staining

The cells were fixed in 4% PFA and permeabilized with 0.05% Triton X-100 (Sigma) in PBS at RT. Non-specific antibody binding sites were blocked with 5% bovine serum albumin (BSA) in PBS for 1 h at 37 °C. The cells were incubated with primary antibodies against NES (Mouse, 1:200, ab22035, Abcam), GFAP (Rabbit, 1:200, ab207165, Abcam), S100B (Mouse, 1:200, S2532, Sigma), and TUBB3 (Rabbit, 1:200, ab133582, Abcam) diluted in 2.5% BSA/PBS overnight at 4 °C. Immunofluorescence labeling was performed via the use of appropriate secondary antibodies conjugated to Alexa Fluor 594 gye (Rabbit, 1:200, 711-585-152, Jackson) and Alexa Fluor 488 gye (Mouse, 1:200, ab150105, Abcam). The nuclei were stained with DAPI (Thermo Fisher Scientific, USA) for 10 min at RT, after which the coverslips were mounted with Dako fluorescence mounting media (S3023, Agilent, USA) and analyzed with a Nikon (Japan) or an Olympus (Japan) microscope.

### RNA sequencing and bioinformatic analysis

RNA sequencing was performed on an Illumina NovaSeq platform (Illumina, San Diego, CA, USA), and the results were analyzed by Shanghai Biochip Co., Ltd., Shanghai, China. The raw data were processed with trim_galore (version 0.6.4). Transcript read counts were determined via HTSeq-count (version 0.13.5), and mRNA expression levels were normalized to FPKM, which represents the number of fragments per kilobase of gene length per million mapped fragments. This normalization accounts for both sequencing depth and gene length effects on fragment counts. Genes with a *P* value <.05 and |FC| > 1.5 were identified as differentially expressed genes. To explore the biological functions of these genes, we conducted enrichment analysis utilizing the GO and KEGG databases, employing the Fisher test for this purpose, which revealed significant enrichment among the differentially expressed genes.

### Crush injury of rat sciatic nerves

The animal experiments complied with the ARRIVE 2.0 guidelines.

Adult male and female specific pathogen-free Sprague-Dawley rats (250-300 g) were used. Animal use was approved under permit SYXK (Hu): 2020-002 and conducted in accordance with the Animal Ethics Committee of Shanghai Jiao Tong University (SH9H-2020-A718-1). Sample sizes were determined using G*Power and the 4R principle; group allocation was randomized (sealed-envelope method) and blinded. Rats were pair-housed, maintained on ad libitum food/water, and kept under a 12 h light/dark cycle.

For DPSC treatment evaluation, 18 rats were randomized into three groups: Sham, PBS, and DPSCs (*n* = 6 each). To assess LARP7’s role in DPSC-mediated nerve repair, 30 rats were randomized into five groups: Sham, PBS, Vector, sh-LARP7, and OE-LARP7 (*n* = 6 each). Exact n values per group are indicated in corresponding figures. Anesthesia was induced with intraperitoneal zoletil 50 (40 mg/kg). After shaving and disinfecting the right thigh with 75% ethanol, a mid-thigh incision exposed the sciatic nerve via blunt dissection of the biceps femoris and superficial gluteus muscles. Nerve injury was induced by compressing the nerve three times (30 s each, 10 s intervals) at ∼6-8 mm distal to the ischial tuberosity using hemostatic forceps calibrated to 5 kg. Injury sites were marked with non-absorbable sutures. The DPSC group received 2 × 10^5^ cells in 10 µL PBS injected locally; the PBS group received vehicle only. In the Sham group, the nerve was exposed and re-covered without injury or injection.

All animals received 10^5^ units of intraperitoneal penicillin post-surgery. Functional recovery was assessed weekly via sciatic functional index (SFI) for 4 weeks; electrophysiology was performed at week 4. At 4 weeks post-injury, rats were euthanized by CO_2_ inhalation, and tissues were harvested for histology—evaluating regenerated nerve architecture and gastrocnemius morphology. Animals experiencing unexpected severe adverse events were excluded, and experiments were repeated as needed.

### Histology

Hematoxylin-eosin (HE) staining was performed according to the manufacturer’s instructions (G1005, Servicebio). In brief, the paraffin sections were dewaxed and stained with hematoxylin for 5 min, followed by dehydration in 85% and 95% ethanol for 5 min each. The samples were subsequently stained with eosin for 5 min, dehydrated in 100% ethanol for an additional 5 min, cleared with xylene for 10 min, mounted with neutral gum, and examined under a microscope.

Immunohistochemical staining for MBP (Rabbit, 1:1000, CST, #78896), NF200 (Rabbit, 1:200, Proteintech, 18934-1-AP) and ERBB4 (Rabbit, 1:100, Proteintech, 19943-1-AP) was performed according to the manufacturer’s instructions (ZSGB-Bio, SP-9000). Briefly, the slides were incubated with primary antibodies overnight at 4 °C, followed by incubation with HRP-conjugated secondary antibodies for 1 h at 37 °C. DAB (ZSGB-Bio, ZLI-9018) was used to visualize the immunocomplexes, and hematoxylin was used to counterstain the nuclei.

For immunofluorescence staining, the following antibodies were used for overnight incubation at 4 °C: S100B (Rabbit, 1:100, Abcam, ab52642), NF200 (Rabbit, 1:200, Proteintech, 18934-1-AP), and NGFR (Rabbit, 1:200, CST, #8238S).

For toluidine blue (TB) staining and transmission electron microscope (TEM), tissue blocks were fixed overnight at 4 °C in 2.5% glutaraldehyde, then post-fixed for 2 h at room temperature in 1% osmium tetroxide. After dehydration, tissues were embedded in epoxy resin and polymerized for >48 h at 65 °C. Semi-thin sections (1.5 μm) were cut using a Leica HistoCore Nanocut R (Germany) and stained with 1% toluidine blue. Myelinated fiber density (fibers/1000 μm^2^) was quantified from six non-overlapping fields per sample. Ultrathin sections (60-80 nm) were prepared with an ultramicrotome, stained with saturated 2% uranyl acetate (in ethanol) and 2.6% lead citrate, and imaged using a Hitachi HT7800 TEM (Japan). All histological and EM processing was performed by Servicebio. Axon and myelinated fiber diameters and myelin sheath thickness were measured using Olympus cellSens Dimension software; the G-ratio was calculated as axon diameter divided by total fiber diameter.

### Functional analysis

The walking track and footprint analysis results were assessed. In summary, rat footprints were obtained by immersing the paws in ink and allowing the rats to traverse an 80 × 11 × 15 cm corridor lined with a sheet of white paper at the bottom. The collected tracts were utilized to compute the SFI based on the basis of the measurements: distance between the first and fifth toes (toe spread, TS), third toe to heel (print length, PL), and second to fourth toes (intermediate toe spread, IT).These indices were measured and calculated on the basis of the experimental legs (E) and normal legs (N) via the following formula^3^:


$$\begin{array}{c} SFI=-38.3\times (\mathrm{EPL}-\mathrm{NPL})/\mathrm{NPL}+109.5\times (\mathrm{ETS}-\mathrm{NTS})/\mathrm{NTS}\\ +13.3\times (\mathrm{EIT}-\mathrm{NIT})/\mathrm{NIT}-8.8 \end{array}$$

The SFI is normally a negative number ranging between −100 and 0. A score of −100 represents non-functional sciatic nerves, whereas 0 indicates normal nerve function or complete nerve recovery. Therefore, a higher number means a better function.

For electrophysiological assessment, animals were anesthetized at 28 days post-surgery, and the sciatic nerve was exposed. Motor nerve action potentials were recorded using an RM6240E multichannel physiological signal acquisition system with the following parameters: scanning speed 2.00 ms/div, sensitivity 1 mV, time constant 0.001 s, notch filter 100 Hz, stimulus intensity 0.4 V, and electrode distance 1 cm.

Four weeks after surgery, bilateral gastrocnemius muscles were harvested and weighed using an electronic balance (BSA822-CW, Sartorius, Germany). The wet weight ratio (injured side/contralateral side) was calculated, with the contralateral uninjured nerve serving as the internal control. Gross images were captured using a digital camera (Canon). Muscles were fixed in 4% PFA, paraffin-embedded, sectioned, and stained with hematoxylin and eosin. Cross-sectional areas were quantified from five randomly selected fields per sample using Image-Pro Plus software.

### Statistics

Statistical analysis was performed via GraphPad Prism v.10.1.2. One-way ANOVA was used to assess the statistical significance of differences among multiple groups, whereas Student’s *t*-test was used to compare two groups. The data are presented as the means ± standard deviations (SDs), with statistical significance set at *P* <.05.

## Results

### DPSCs exhibit multilineage neural differentiation potential and promote peripheral nerve regeneration via Schwann cell-like differentiation

**Figure 1.**
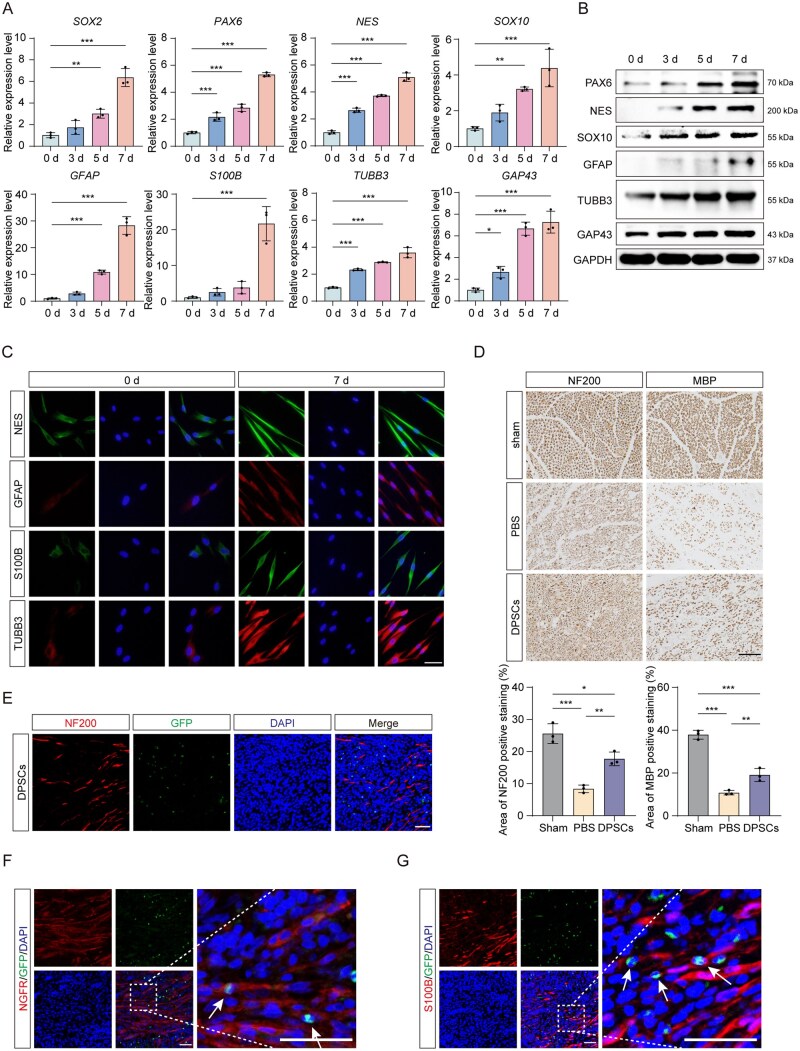
DPSCs contribute to peripheral nerve repair via differentiating into Schwann cells. (A and B) The mRNA expression levels of *SOX2, PAX6, NES, SOX10*, *GFAP, S100B, TUBB3,* and *GAP43* were evaluated by qRT-PCR (*n* = 3), and the protein expression levels of NES, PAX6, SOX10, GFAP, TUBB3, and GAP43 were evaluated by WB after neural induction for 0-7 d. (C) Immunofluorescence assay showing the expression of NES, GFAP, S100B, and TUBB3 after neural induction of DPSCs for 0-7 d. Nuclei were stained with DAPI (blue). Scale bars, 20 μm. (D) NF200 and MBP expression in the cross-sections of sciatic nerves are presented in the images. Scale bars, 100 μm. The expression levels of NF200 and MBP detected by immunohistochemistry were statistically quantified (*n* = 3). (E-G) Representative images showing DPSC-GFP and (E) NF200, (F) NGFR, and (G) S100B fluorochrome labels in the longitudinal sections of sciatic nerves. Nuclei were stained with DAPI. Scale bars, 50 μm. The full-length blots are presented in Figure S4. ^*^P <.05, ^**^P <.01, ^***^P <.001. The data are presented as the means ± SDs.

The isolated DPSCs presented characteristic mesenchymal stem cell properties (Figure S1). To assess their neurogenic capacity, DPSCs underwent neural induction for 7 days, resulting in significant upregulation of neural progenitor markers (SOX2, PAX6, and NES), Schwann cell markers (SOX10, GFAP, and S100B), and neuronal markers (TUBB3 and GAP43) (Figure 1A and B). Immunofluorescence analysis revealed morphological transformation from a fibroblastic phenotype to a neural-like phenotype (cellular elongation and process formation) in differentiated DPSCs (Figure 1C). This coincided with increased expression of neural lineage markers compared with undifferentiated controls, confirming the neurogenic potential of DPSCs under suitable inductive conditions.

To evaluate the therapeutic potential of DPSCs in PNIs, DPSCs were transplanted into crushed rat sciatic nerves. Four weeks post-surgery, the NF200- and MBP-positive areas in the DPSCs group were markedly greater than those in the PBS group but remained lower than those in the Sham group (Figure 1D). These findings indicate that DPSCs contribute to nerve recovery, although the structural restoration remained incomplete compared with that in sham-operated animals. To trace the fate of the transplanted cells, GFP-labeled DPSCs were transplanted into injury sites. Two weeks post-transplantation, the transplanted DPSCs partially expressed Schwann cell markers (NGFR and S100B) but not a neuronal marker (NF200) (Figure 1E and G), indicating that DPSCs contribute to nerve repair primarily through differentiation toward Schwann cell-like phenotypes.

### LARP7 promotes Schwann cell-like differentiation of DPSCs with minimal induction toward alternative neural lineages

**Figure 2.**
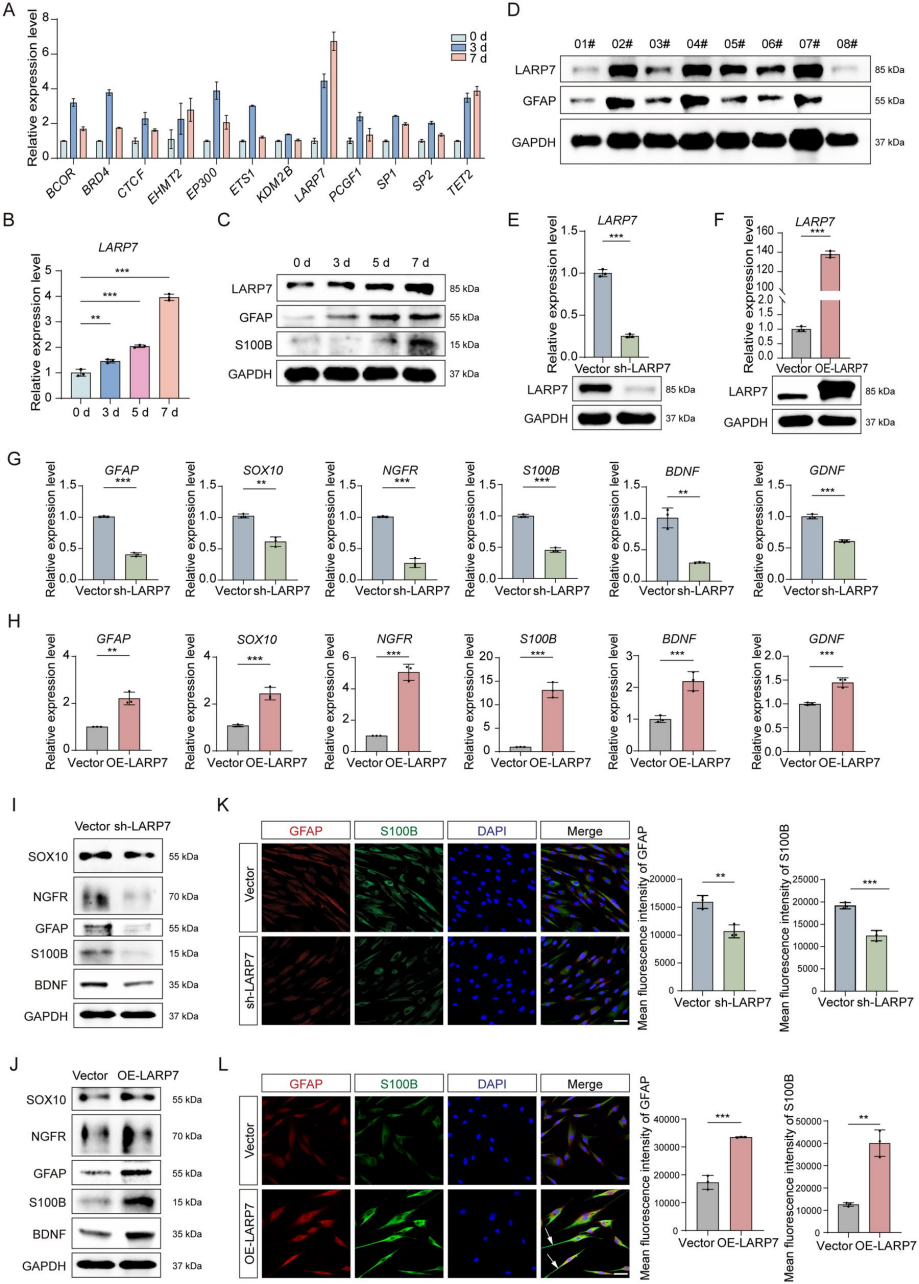
LARP7 promotes the differentiation of DPSCs into Schwann cell-like phenotypes. (A) The mRNA expression of a panel of genes associated with neurodevelopment and neural differentiation during the neural differentiation of DPSCs for 0-7 d was evaluated by qRT-PCR (*n* = 3). (B and C) The mRNA expression levels of *LARP7* were evaluated by qRT-PCR, and the protein expression levels of LARP7, GFAP, and S100B were evaluated by WB after neural induction for 0-7 d. (D) WB analysis of the protein expression levels of LARP7 and GFAP in different individuals. (E-L) LARP7 expression was changed via lentiviral transduction, DPSCs were treated with neural induction medium for 3 days. (E, F) The mRNA expression level of LARP7 was evaluated by qRT-PCR (*n* = 3), and the protein expression level was evaluated by WB. (G, H) The mRNA expression levels of *GFAP*, *SOX10*, *NGFR*, *S100B*, *BDNF*, and *GDNF* were evaluated by qRT-PCR (*n* = 3), and (I, J) the protein expression levels of GFAP, SOX10, NGFR, S100B, and BDNF were evaluated by WB. (K, L) Immunofluorescence assay and the corresponding statistical analyses revealed the protein expression of GFAP and S100B. Nuclei were stained with DAPI. Scale bars, 50 μm. The full-length blots are presented in Figure S5. ^*^P < .05, ^**^P <.01, ^***^P <.001. The data are presented as the means ± SDs.

To identify regulatory factors underlying the therapeutic potential of DPSCs in PNIs, we analyzed gene expression changes in neurodevelopment- and neural differentiation-related genes during DPSC neural differentiation. Notably, LARP7 expression increased markedly during DPSC neural differentiation (Figure 2A-C). Additionally, there was a positive correlation was identified between LARP7 and GFAP (Schwann cell marker) among different individuals (Figure 2D). Lentivirus-mediated knockdown and overexpression of LARP7 in DPSCs (Figure 2E and F) revealed that LARP7 depletion decreased the expression of Schwann cell markers and neurotrophic factors, whereas LARP7 overexpression increased their expression (Figure 2G-J). In contrast, LARP7 had limited effects on other neural lineages (data not shown). Moreover, LARP7-overexpressing DPSCs presented increased GFAP and S100B levels, with elongated protrusions and bipolar/multipolar morphology resembling Schwann cells (Figure 2K and L). These findings suggest that LARP7 positively regulates the Schwann cell-like differentiation of DPSCs and enhances their neurotrophic effects.

### RNA-Seq reveals that cytokine-cytokine receptor interaction and the PI3K-Akt signaling pathway are involved in LARP7-mediated DPSC changes

**Figure 3.**
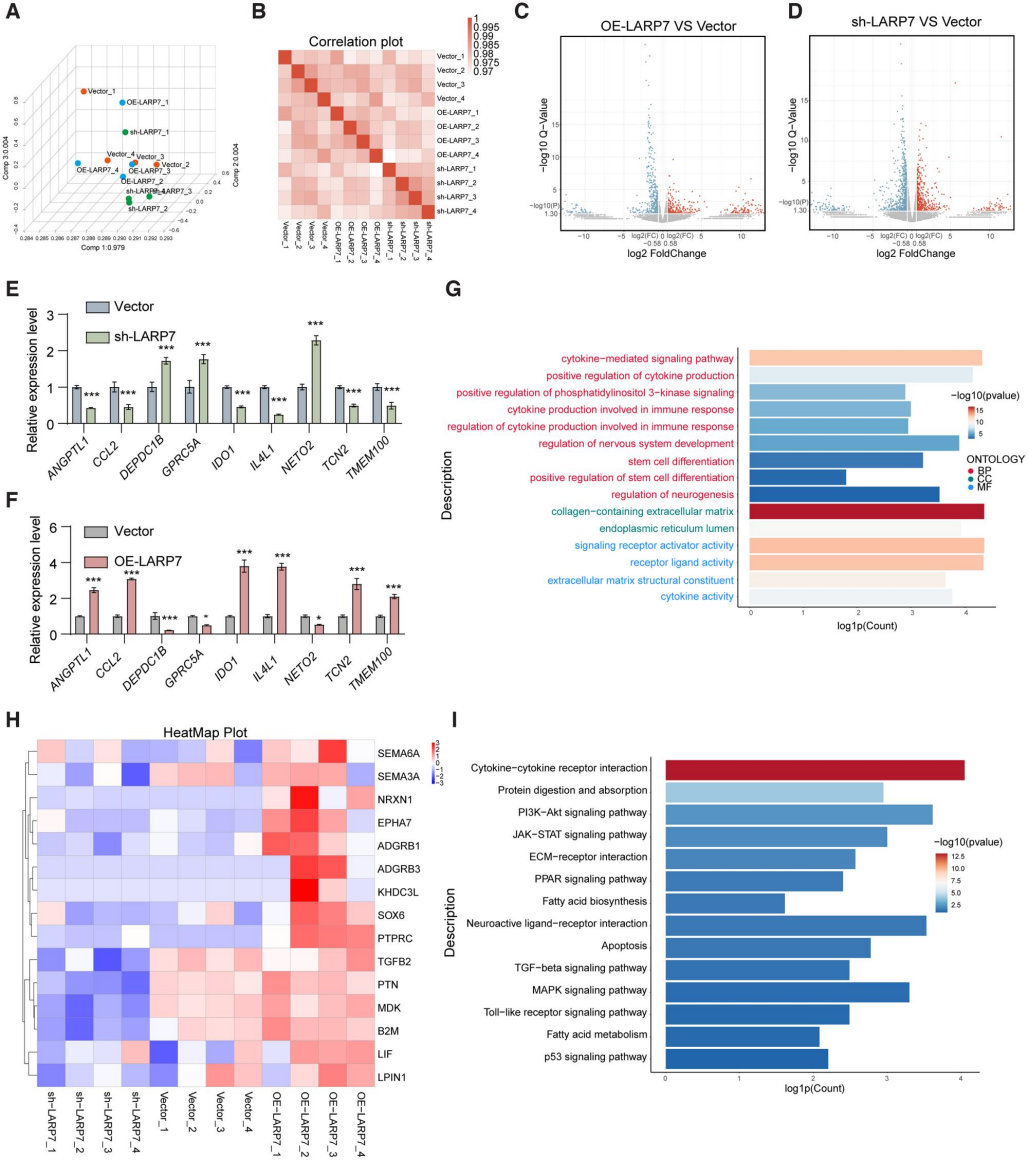
RNA-Seq reveals that cytokine-cytokine receptor interaction and the PI3K-Akt signaling pathway are involved in LARP7-mediated DPSC changes. RNA sequences were used to analyze the whole genome gene expression of DPSCs transfected with lentiviruses with different LARP7 expression levels. (A, B) Principal component analysis (PCA) and Pearson correlations of each group. (C, D) Volcano plot of differentially expressed genes of sh-LARP7 vs Vector and OE-LARP7 vs Vector. (E, F) The mRNA expression levels were evaluated by qRT-PCR (*n* = 3). (G) GO biological process terms enriched in datasets of genes downregulated by LARP7 knockdown and upregulated by LARP7 overexpression. (H) Heatmap of the differentially expressed genes in different groups. RNA-seq analysis of the sh-LARP7, vector, and OE-LARP7 populations revealed significantly differentially expressed genes in these populations. Cutoff line: Fold change >1.5 and adjusted *P* value of <.05. (I) KEGG analysis of the datasets of genes whose expression was affected by LARP7 knockdown and whose expression was upregulated by LARP7 overexpression.

To explore the molecular mechanisms underlying LARP7-mediated changes, we analyzed whole-genome expression in DPSCs with altered LARP7 levels. Principal component analysis and Pearson correlations confirmed the data suitability (Figure 3A and B). LARP7 overexpression upregulated 262 genes and downregulated 452 genes; LARP7 knockdown upregulated 575 genes and downregulated 696 genes (Figure 3C and D). qRT-PCR validation of nine genes confirmed the reliability of the RNA-seq results (Figure 3E and F). Gene Ontology (GO) analysis revealed enrichment in the regulation of cytokine production, phosphatidylinositol 3-kinase signaling, nervous system development, and stem cell differentiation (Figure 3G). The cellular component terms included “collagen-containing extracellular matrix” and “molecular functions involved signaling receptor activator activity” (Figure 3G). Heatmaps revealed that LARP7 significantly affected genes related to nervous system development and stem cell differentiation (*SEMA6A*, *NRXN1*, *SOX6*, *EPHA7*, *ADGRB1*, *SEMA3A*, etc.) (Figure 3H). Kyoto Encyclopedia of Genes and Genomes (KEGG) analysis highlighted cytokine-cytokine receptor interactions and the PI3K-Akt signaling pathway (Figure 3I). These results indicate that LARP7 may regulate the cytokine secretion and differentiation of DPSCs via these pathways.

### LARP7 enhances the trophic/anti-inflammatory effects of DPSCs via cytokine-cytokine receptor interactions and promotes the Schwann cell-like differentiation of DPSCs via the PI3K-Akt-mTOR pathway

**Figure 4.**
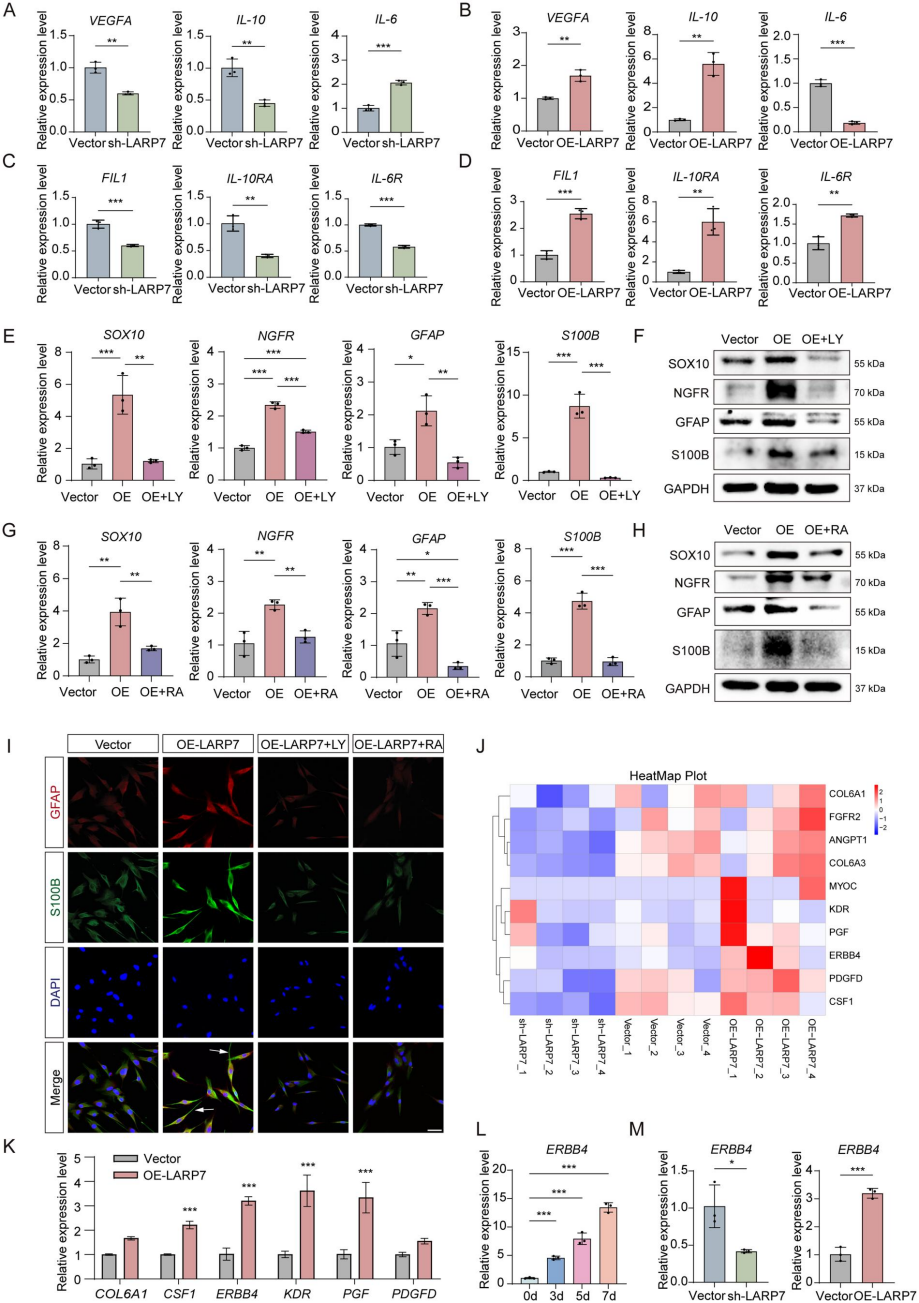
LARP7 enhances the trophic/anti-inflammatory effects of DPSCs via cytokine-cytokine receptor interactions and promotes the Schwann cell-like differentiation of DPSCs via the PI3K-Akt-mTOR pathway. (A-D) After transfection with lentivirus to intervene the expression of LARP7, DPSCs were treated with neural induction media for 3 days. (A, B) The mRNA expression levels of *VEGF, IL-10, and IL-6* were evaluated by qRT-PCR (*n* = 3). (C, D) The mRNA expression levels of *FIL1, IL-10RA, and IL-6R* were evaluated by qRT-PCR (*n* = 3). (E-I) After transfection with lentivirus to affect the expression of LARP7, DPSCs were treated with neural induction medium and LY294002 (a PI3K-Akt signaling inhibitor, 25 μM) or Rapamycin (an mTOR signaling inhibitor, 200 nM) for 3 days. (E, G) The mRNA expression levels of *GFAP*, *SOX10*, *NGFR, and S100B* were evaluated by qRT-PCR (*n* = 3), and (F, H) the protein expression levels of GFAP, SOX10, NGFR, and S100B were evaluated by WB. (I) Immunofluorescence showing that protein expression of GFAP and S100B. Nuclei were stained with DAPI. Scale bars, 50 μm. (J) Heatmap of the differentially expressed genes in the PI3K-Akt signaling pathway. (K) The mRNA expression levels of *COL6A1*, *CSF1*, *ERBB4, KDR, PGF, and PDGFD* were evaluated by qRT-PCR (*n* = 3). (L) The mRNA expression levels of *ERBB4* were evaluated by qRT-PCR after neural induction for 0-7 d (*n* = 3). (M) LARP7 expression was altered via lentiviral transduction. The mRNA expression level of *ERBB4* was evaluated by qRT-PCR (*n* = 3). The full-length blots are presented in Figure S6. ^*^P <.05, ^**^P <.01, ^***^P <.001. The data are presented as the means ± SDs.

To assess the role of the cytokine-cytokine receptor interaction signaling in LARP7-mediated effects, we examined the expression of several related cytokines and their receptors. LARP7 knockdown reduced *VEGFA* (angiogenic growth factor), *FIL1* (VEGFA receptor), *IL-10* (anti-inflammatory factor), *IL-10RA* (IL-10 receptor) and *IL-6R* (IL-6 receptor) expression, while increasing *IL-6* (pro-inflammatory factor) levels (Figure 4A and C). In contrast, LARP7 overexpression had the opposite effect (Figure 4B and D).

To evaluate the involvement of the PI3K-Akt pathway in LARP7-mediated effects, we treated DPSCs with LY294002, a PI3K inhibitor. LY294002 suppressed PI3K-Akt and downstream signaling mTOR activation induced by LARP7 overexpression (Figure S2A). It also reduced the increase the expression of Schwann cell markers (Figure 4E and F). mTOR is a key kinase in the downstream signaling of the PI3K-Akt pathway and has been reported to be relevant to Schwann cell differentiation.^29^ Given that mTOR is activated by LARP7, we further tested its role using rapamycin (an mTOR inhibitor). Rapamycin inhibited mTOR activation and reduced Schwann cell marker expression (Figure S2B; Figure 4G and H). Immunofluorescence for GFAP and S100B revealed that LY294002 and rapamycin caused LARP7-overexpressing DPSCs to adopt a fibroblast-like morphology instead of a bipolar/multipolar Schwann cell-like shape (Figure 4I). Heatmaps revealed the differential expression of genes in the PI3K-Akt pathway (Figure 4J). qRT-PCR of six selected genes revealed significant upregulation of *ERBB4* (Figure 4K), which was also elevated during DPSC neural differentiation (Figure 4L). Manipulating LARP7 expression revealed a positive correlation between ERBB4 and LARP7 levels (Figure 4M), suggesting that ERBB4 is a potential downstream effector of LARP7.

### ERBB4 inhibition attenuates LARP7-mediated Schwann cell differentiation in DPSCs

**Figure 5.**
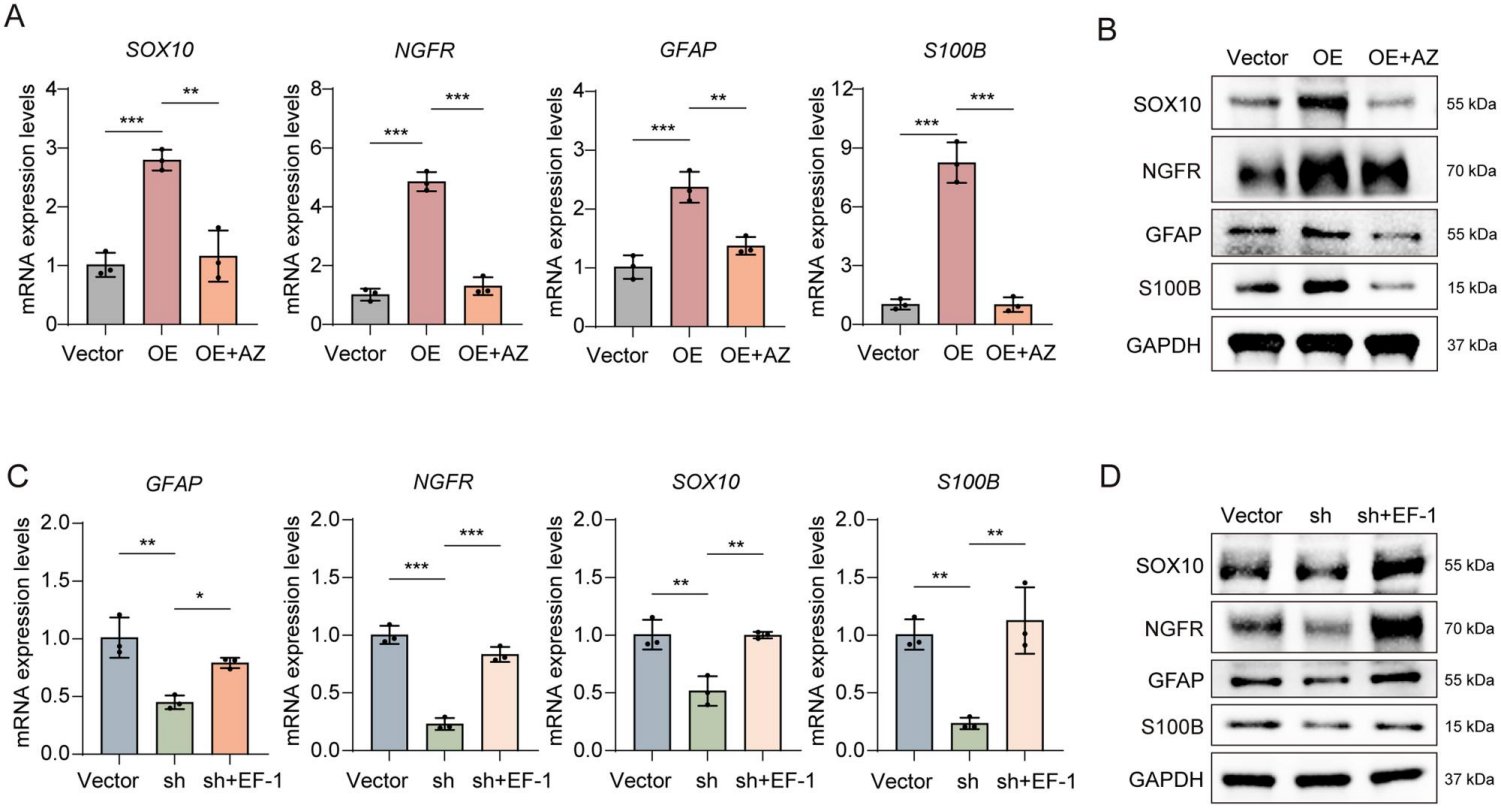
ERBB4 inhibition attenuates LARP7-mediated Schwann cell differentiation in DPSCs. (A and B) Following lentiviral-mediated LARP7 overexpression, DPSCs were subjected to neural induction for 3 days in the presence of AZ-5104 (an ERBB4 inhibitor, 10 nM). (A) The mRNA expression levels of *GFAP, SOX10, NGFR, and S100B* were evaluated by qRT-PCR (*n* = 3). (B) The protein expression levels of GFAP, SOX10, NGFR, and S100B were evaluated by WB. (C and D) Conversely, following lentiviral-mediated LARP7 knockdown, DPSCs underwent the same 3-day neural induction in the presence of EF-1 (an ERBB4 agonist, 10 μM). (C) The mRNA expression levels of *GFAP, SOX10, NGFR, and S100B* were evaluated by qRT-PCR (*n* = 3). (D) The protein expression levels of GFAP, SOX10, NGFR, and S100B were evaluated by WB. The full-length blots are presented in Figure S7. ^*^P < .05, ^**^P < .01, ^***^P < .001. The data are presented as the means ± SDs.

To determine whether ERBB4 functions downstream of LARP7, we performed complementary pharmacological interventions in DPSCs with modulated LARP7 expression. Treatment of LARP7-overexpressing DPSCs with the ERBB4 inhibitor AZ-5104 not only reversed the upregulation of Schwann cell markers but also attenuated PI3K-Akt-mTOR signaling (Figure 5A and B; Figure S2C). Conversely, activation of ERBB4 with EF-1 in LARP7-knockdown DPSCs restored both Schwann cell marker expression and pathway phosphorylation (Figure 5C and D; Figure S2D). These findings position ERBB4 as a critical downstream effector through which LARP7 orchestrates Schwann cell-like differentiation via the PI3K-Akt-mTOR cascade.

### LARP7 augments the therapeutic efficacy of DPSCs in rats with PNIs

**Figure 6.**
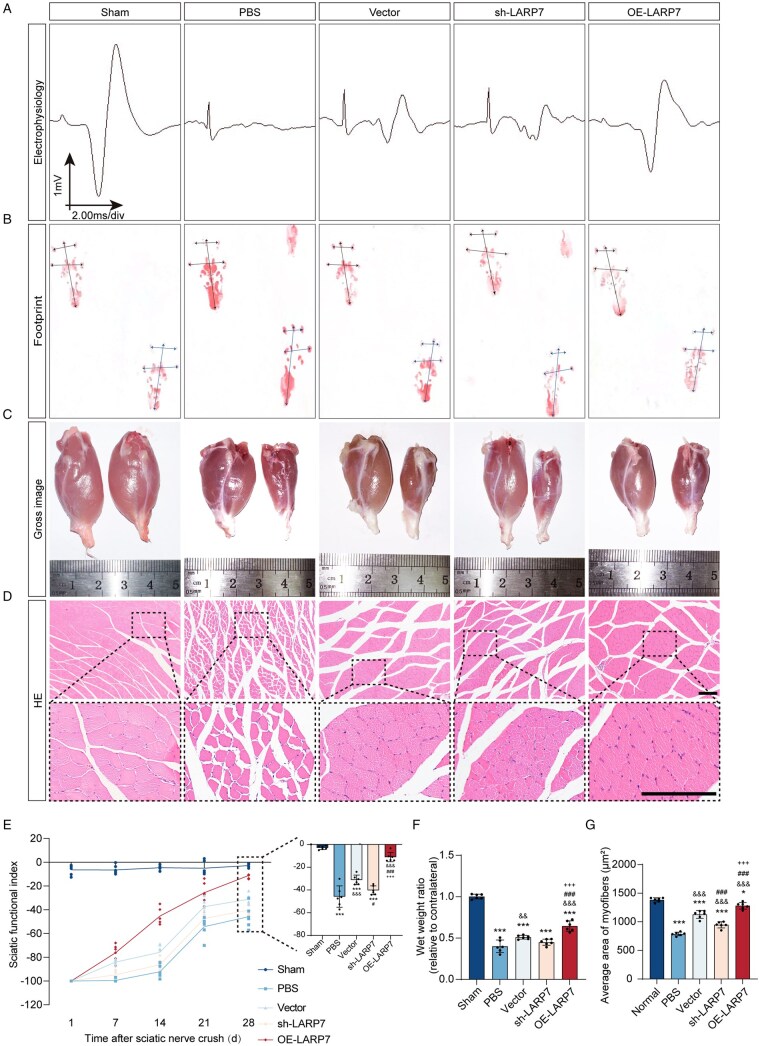
LARP7 improves the therapeutic efficacy of DPSCs in promoting functional recovery in rats with PNIs. At 28 days post-injury and implantation, the functional recovery of injured nerves was analyzed. (A) Electrophysiological examination of injured sciatic nerves. (B) Measurement of footprints. (C) Gross images of the gastrocnemius muscle. (D) HE stained images of gastrocnemius muscle cross-sections. Scale bars, 100 μm. (E) The sciatic nerve function indices (SFIs) from postoperative day 1 to day 28 and the SFI on day 28 are displayed independently for enhanced clarity (*n* = 6). (F) The wet weight ratio of the gastrocnemius muscle on the injured side relative to that on the contralateral side (*n* = 6). (G) The cross-sectional areas of the gastrocnemius fibers detected via HE staining images of the gastrocnemius muscle cross-sections was statistically quantified (*n* = 6). For all charts, ^*^each group vs Sham group. ^&^each group vs PBS group. ^#^each group vs Vector group. ^+^each group vs sh-LARP7 group. ^*^P <.05, ^**^P <.01, ^***^P <.001. ^&^*P* <.05, ^&&^*P* <.01, ^&&&^*P* <.001. ^#^*P* <.05, ^##^*P* <.01, ^###^*P* <.001. ^+^*P* <.05, ^++^*P* <.01, ^+++^*P* <.001. The data are presented as the means ± SDs.

Transfected DPSCs were implanted into rats with crushed sciatic nerves to evaluate whether LARP7 influences their ability to promote nerve repair. Functional recovery is a critical indicator of nerve regeneration.^3^ At 28 days post-surgery, representative compound muscle action potentials (CMAPs) presented the lowest peak amplitude in the PBS group, whereas the OE-LARP7 group presented significantly greater amplitudes than the Vector and sh-LARP7 groups did (Figure 6A). Footprint analysis in rats was used to assess functional recovery, revealing increased footprint length and reduced toe spread in injured animals (Figure 6B). All surgical groups showed partial recovery by day 28, with the sh-LARP7 group having the lowest SFI value and the OE-LARP7 group the highest SFI value (Figure 6E). Delayed nerve recovery leads to gastrocnemius muscle atrophy.^3^ Consistent with this mechanism, the muscle weight ratios revealed that the OE-LARP7 group exhibited significantly less atrophy than the Vector and sh-LARP7 groups did (Figure 6C and F). Furthermore, histological analysis revealed improved muscle fiber structure in the treatment groups compared with the PBS group. Notably, the OE-LARP7 group had larger average muscle fiber areas than the sh-LARP7 group did, while the Vector group showed partial improvement (Figure 6G). These results indicate that LARP7-overexpressing DPSCs promote the functional recovery of peripheral nerves.

**Figure 7.**
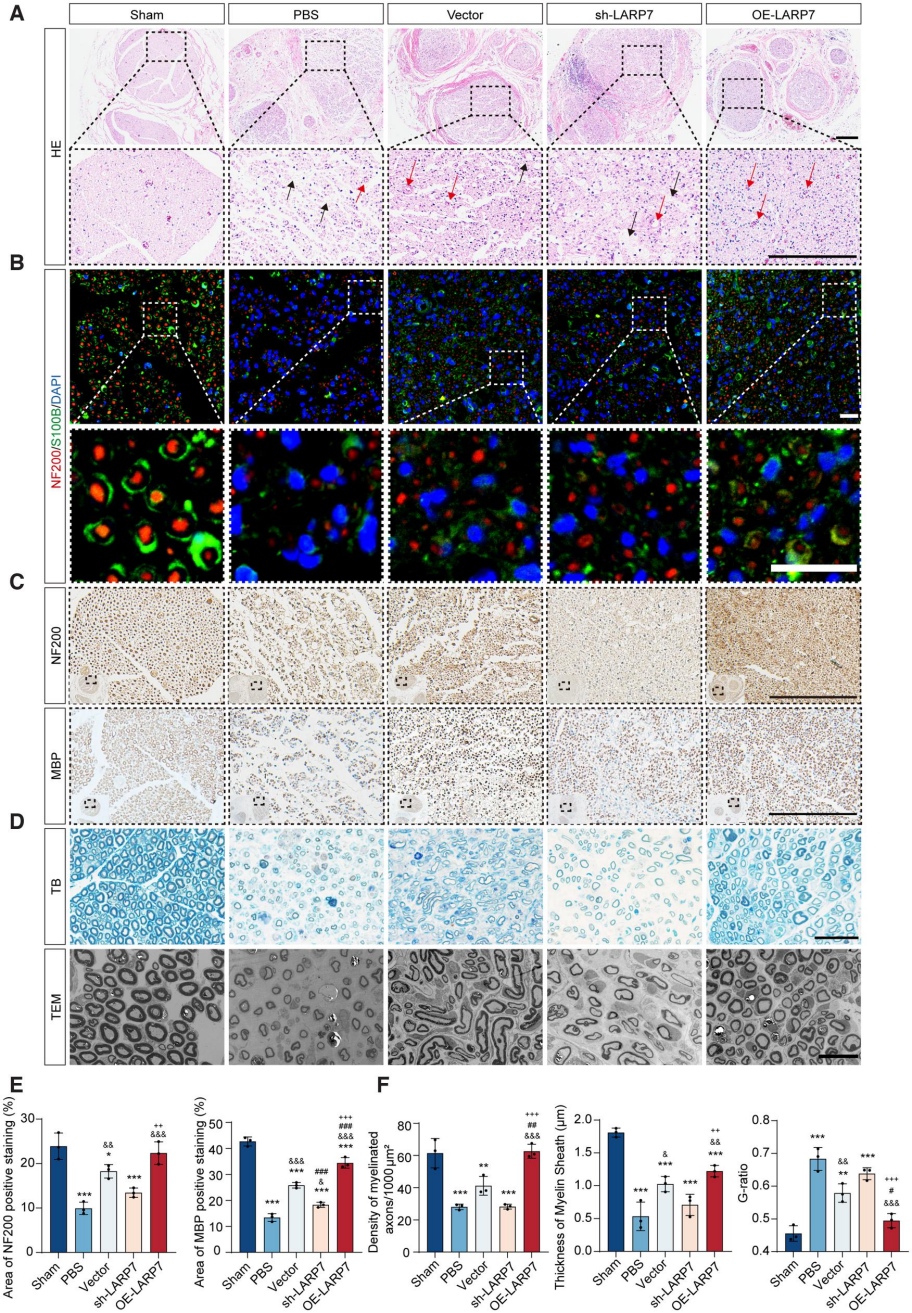
LARP7 increases the therapeutic efficacy of DPSCs in promoting structural regeneration in rats with PNIs. The repaired nerves were examined 28 days after surgery and implantation. (A) Representative images of HE-stained cross-sections of nerves are presented. Cellular edema (black arrows), and blood vessels (red arrows) are shown. Scale bars, 100 μm. (B) Representative images showing NF200 and S100B fluorochrome labels in cross-sections of sciatic nerves. Nuclei were stained with DAPI. Scale bars, 25 μm. (C) NF200 and MBP expressed in the cross-sections of sciatic nerves are presented in the images. Scale bars, 100 μm. (D) Representative images showing TB staining and TEM images of the cross-sections of sciatic nerves. Scale bars, 20 μm. (E) The expression levels of NF200 and MBP detected by immunohistochemistry were statistically quantify (*n* = 3). (F) The mean density of myelinated nerve fibers, the mean thickness of the myelin sheath, and the G-ratio in TB-stained and TEM images (*n* = 3). For all charts, ^*^each group vs Sham group. ^&^each group vs PBS group. ^#^each group vs Vector group. ^+^each group vs sh-LARP7 group. ^*^P < .05, ^**^P <.01, ^***^P <.001. ^&^*P* <.05, ^&&^*P* <.01, ^&&&^*P* <.001. ^#^*P* <.05, ^##^*P* <.01, ^###^*P* <.001. ^+^*P* <.05, ^++^*P* <.01, ^+++^*P* <.001. The data are presented as the means ± SDs.

HE staining was used to evaluate nerve structural recovery and revealed significant cellular edema and inflammatory infiltration in the PBS and sh-LARP7 groups, whereas the Vector and OE-LARP7 groups presented reduced tissue damage (Figure 7A). The OE-LARP7 group also presented increased blood vessel formation, which supports nerve repair (Figure 7A). Brain-derived neurotrophic factor (BDNF), secreted by Schwann cells, functions as a positive regulator of myelination.^30^ We thus examined the effect of LARP7-modified DPSCs on BDNF expression in the repairing nerves. Importantly, BDNF levels were reduced in the sh-LARP7 group and increased in the OE-LARP7 group (Figure S3A and B). These results suggest that LARP7-overexpressing DPSCs exhibit enhanced trophic support and anti-inflammatory capabilities.

Triple staining of the sciatic nerve cross-sections was subsequently performed via NF200 and S100B to visualize their characteristic structures. The Sham group presented well-organized axons (NF200, red) surrounded by myelin sheaths (S100B, green), whereas the PBS group presented disorganized and reduced signals. DPSC transplantation improved axon and myelin structure, especially in the OE-LARP7 group (Figure 7B). Further analysis via NF200 and MBP staining showed that LARP7 knockdown reduced the expression of axon and myelin markers, whereas LARP7 overexpression increased the expression of these markers (Figure 7C and E). Schwann cells promote functional recovery by myelinating axons to increase the conduction velocity and maintain axonal integrity.^31^^,^^32^ TB staining and TEM were used to further evaluate myelination, and the results revealed that LARP7 knockdown decreased myelinated fiber density and myelin thickness while increasing the G-ratio, indicating impaired remyelination. In contrast, LARP7 overexpression improved these parameters (Figure 7D and F). To corroborate the functional involvement of ERBB4 in *vivo*, we assessed ERBB4 expression in regenerating nerve specimens from each treatment group. Immunohistochemical staining demonstrated a marked increase in ERBB4 expression in the OE-LARP7 group relative to the other treatment groups (Figure S3C and D). These results demonstrate that LARP7 enhances the capacity of DPSCs to promote structural recovery of peripheral nerves.

## Discussion

Although the peripheral nervous system possesses regenerative capacity, functional recovery from PNIs remains a significant clinical challenge due to insufficient endogenous repair mechanisms.^33^^,^^34^ DPSCs have emerged as promising candidates for cell-based nerve repair strategies^35^; however, their efficacy is limited by microenvironmental constraints and cellular heterogeneity.^24^ Genetic modification of stem cells represents a viable approach to enhance their therapeutic potential.^25^ In this study, we demonstrated that LARP7 enhances the therapeutic efficacy of DPSCs by increasing their trophic and anti-inflammatory effects through cytokine-cytokine receptor interactions and promoting their Schwann cell-like differentiation via the activation of the PI3K-Akt-mTOR signaling pathway, with ERBB4 serving as a critical downstream effector, thereby collectively facilitating peripheral nerve repair.

DPSCs mediate repair through differentiation and replenishment at injury sites and secretion of cytokines that provide trophic support and anti-inflammatory activity.^36^^,^^37^ Importantly, transplanted DPSCs contributed to enhanced peripheral nerve repair and expressed Schwann cell markers at the injury sites, supporting previous findings.^38^ These findings suggest that DPSCs primarily differentiate into Schwann cells in the repair of PNIs, thereby reinforcing our focus on Schwann cell differentiation in future investigations. Studies have reported that DPSCs possess intrinsic neurogenic potential and are capable of differentiating into both neurons and glial cells.^39^^,^^40^ However, a universally standardized in vitro induction protocol has not yet been established. In this study, the neural induction medium we employed is based on neurobasal medium supplemented with B-27, basic fibroblast growth factor (bFGF), and epidermal growth factor (EGF). Previous studies have shown that EGF promotes neuronal differentiation and that bFGF drives Schwann cell specification in neural crest stem cells, while their co-administration favors neuronal commitment.^41–43^ However, our results revealed that induced DPSCs presented significantly elevated expression of multiple neural markers, including Schwann cell-specific markers. On the basis of these findings, we propose that this formulation creates a permissive neural microenvironment without imposing lineage restriction. Thus, our protocol effectively promotes neural differentiation in DPSCs without significant lineage bias, offering a suitable model for studying Schwann cell-like differentiation.

LARP7, a member of the evolutionarily conserved La-related protein family of RNA-binding proteins that regulate RNA processing, maturation, and translational control,^44^^,^^45^ has been implicated in neurodevelopment. We observed a positive correlation between individual variations in LARP7 expression and neural markers, suggesting a significant association between LARP7 and the neurogenic potential of DPSCs. Further evidence demonstrated that LARP7 promotes the differentiation of DPSCs into Schwann cell-like phenotypes and enhances peripheral nerve repair. Schwann cells facilitate repair by upregulating trophic factors (eg, BDNF and VEGF) that support neuronal survival, promote axonal elongation, and stimulate neovascularization.^46^ The neurovascular unit further enables regeneration by providing metabolic support through perfusion, facilitating neurotrophic signaling, and serving as a structural scaffold for Schwann cell and axon migration.^47^^,^^48^ Importantly, we demonstrated that LARP7 upregulated the expression of angiogenesis-related factors (eg, *VEGFA*), anti-inflammatory cytokines (eg, *IL-10*), and their corresponding receptors in DPSCs, thereby enhancing their trophic and immunomodulatory functions. These findings support previous reports showing that LARP7 enhances angiogenic capacity and alleviates oxidative stress-induced damage.^49^ In *vivo* studies further confirmed that LARP7-modified DPSCs promoted neovascularization, reduced inflammatory responses, increased BDNF expression, and improved remyelination.

Our KEGG analysis revealed significant enrichment of LARP7-mediated changes in the PI3K-Akt signaling pathway. Phosphatidylinositol 3 kinases (PI3Ks), their downstream mediators Akt and mammalian target of rapamycin (mTOR) constitute the core components of the PI3K-Akt-mTOR signaling cascade and regulate cell proliferation, survival, development, and differentiation.^50^ This pathway is established as a key regulator of glial differentiation, with pharmacological inhibition of Akt shown to impair Schwann cell differentiation in adipose-derived stem cells.^51–53^ Consistent with these reports, LY294002 (a PI3K/Akt inhibitor) and rapamycin (an mTOR inhibitor) abolished both LARP7-mediated phosphorylation of the PI3K-Akt-mTOR axis and DPSC differentiation into Schwann cell-like phenotypes.

Erb-b2 receptor tyrosine kinase 4 (ERBB4), a member of the epidermal growth factor receptor family, transduces neuregulin signaling to regulate cellular differentiation and proliferation and plays well-established roles in nervous system development.^54^^,^^55^ The Neuregulin-1 (NRG1)/ErbB signaling network is critically involved in Schwann cell development, survival, and myelination in the peripheral nervous system.^56^^,^^57^ In our study, ERBB4 expression was upregulated during DPSC neural differentiation and positively correlated with LARP7 expression levels. Through pharmacological inhibition and *in vivo* expression analysis, we further demonstrated that ERBB4 inhibition reversed LARP7 overexpression-induced upregulation of Schwann cell markers and PI3K-Akt-mTOR signaling, while ERBB4 activation rescued these effects in LARP7-knockdown DPSCs. These findings provide strong evidence that ERBB4 is not merely correlated with but is functionally required for LARP7-mediated Schwann cell-like differentiation of DPSCs. Furthermore, the observation that ERBB4 modulates PI3K-Akt-mTOR signaling downstream of LARP7 positions ERBB4 as a critical signaling node in this regulatory cascade, consistent with the established role of NRG1/ErbB signaling in activating PI3K-Akt pathways during Schwann cell development.^57^ The in *vivo* relevance of this mechanism was further supported by our observation that LARP7-overexpressing DPSC transplantation resulted in significantly higher ERBB4 expression at the injury site in the rat sciatic nerve crush model. Future studies utilizing *Erbb4* knockout models or pharmacological inhibition of ERBB4 in conjunction with DPSC transplantation would be necessary to definitively establish whether ERBB4 is required for the therapeutic effects observed with LARP7-overexpressing DPSCs.

Clinical translation of our findings requires addressing safety concerns associated with viral vectors, including insertional mutagenesis and immunogenicity.^58^ Alternative strategies to safely modulate LARP7 or ERBB4 expression include small molecules that upregulate endogenous expression, non-viral delivery systems (eg, nanoparticles, mRNA) enabling transient expression without genomic integration, and protein-based therapies delivering recombinant factors directly to injury sites.^59^^,^^60^ Notably, SB623, an allogeneic bone marrow-derived MSC product modified with a plasmid encoding the intracellular domain of human Notch1 using lipofectamine transfection, has successfully completed Phase II clinical trials for chronic motor deficits from traumatic brain injury and ischemic stroke, demonstrating the feasibility of non-viral genetically modified cell therapies in neurological indications.^61–63^ Preconditioning strategies that transiently enhance LARP7 expression prior to transplantation or small-molecule LARP7 upregulation offer additional non-genetic approaches to boost therapeutic efficacy. Future research should focus on developing and validating such approaches to facilitate the clinical translation of LARP7-enhanced DPSC therapies, thereby significantly improving the clinical feasibility and safety profile of DPSC-based nerve repair.

## Conclusion

In summary, we identify LARP7 as a novel regulator of DPSCs-mediated PNIs repair. Transplantation of LARP7-overexpressing DPSCs significantly enhanced functional and structural recovery in rats. Mechanistically, LARP7 potentiates DPSC-based therapy by promoting trophic and anti-inflammatory effects via cytokine-cytokine receptor interactions and driving Schwann cell-like differentiation through the PI3K-Akt-mTOR pathway, with ERBB4 as a critical downstream effector. While viral gene modification confirms proof-of-concept, future translation should prioritize non-viral strategies, such as small molecules or recombinant proteins, to address safety concerns. The LARP7-ERBB4 axis thus represents a promising target for optimizing DPSC-based therapies for peripheral nerve regeneration.

## Supplementary Material

sxag013_Supplementary_Data

## Data Availability

The data underlying this article will be shared on reasonable request to the corresponding author.
