# Supplementary material for: LARP7 enhances the potential of dental pulp stem cells to promote peripheral nerve repair
Source: Stem Cells. 2026 Mar 11;44(6):sxag013. doi: 10.1093/stmcls/sxag013 (PMC13223745; doi:10.1093/stmcls/sxag013)
Supplement: sxag013_Supplementary_Data [file sxag013_supplementary_data.docx]

**Supplementary Material**

**Running head: LARP7 Boosts DPSC-Mediated Peripheral Nerve Repair**

**LARP7 Enhances the Potential of Dental Pulp Stem Cells**

**to Promote Peripheral Nerve Repair**

Zihan Yang^1,7^, Guanlin Qu^1,7^, Xiping Wang^2^, Li Wang^1,2^, Lu Chen^1^, Guiqiang Fu^3^, Wenze Chen^3^, Zitong Yang^4^, Wenjing Li^1^, Yuqiong Zhou^1^, Jiacheng Jin^5^, Linxi Zhou^6,*^ and Duohong Zou^1,*^

^1^Department of Oral Surgery, Shanghai Ninth People's Hospital, Shanghai Jiao Tong University School of Medicine; College of Stomatology, Shanghai Jiao Tong University; National Center for Stomatology; National Clinical Research Center for Oral Diseases; Shanghai Key Laboratory of Stomatology, Shanghai, China, 200001

^2^Institute of Stomatology, School and Hospital of Stomatology, Wenzhou Medical University, Wenzhou 325027, China.

^3^Stomatology Hospital and College, Key Laboratory of Oral Diseases Research of Anhui Province, Anhui Medical University, Hefei, China, 230000

^4^Zhejiang Chinese Medical University School of Medical Technology and Information Engineering, Hangzhou, China, 310053

^5^Touro College of Dental Medicine at New York Medical College, Hawthorne, New York, USA, 10532

^6^Department of Orthodontics, Shanghai Ninth People's Hospital, Shanghai Jiao Tong University School of Medicine; College of Stomatology, Shanghai Jiao Tong University; National Center for Stomatology; National Clinical Research Center for Oral Diseases; Shanghai Key Laboratory of Stomatology, Shanghai, China, 200001

^7^Authors contributing equally to this article

Zihan Yang: Conception and design, Collection and/or assembly of data, Data analysis and interpretation, manuscript writing, final approval of manuscript

Guanlin Qu: Conception and design, financial support, data analysis and interpretation, manuscript writing, final approval of manuscript

Xiping Wang: Financial support, data analysis and interpretation, manuscript writing, final approval of manuscript

Li Wang: Collection and/or assembly of data, data analysis and interpretation, manuscript writing, final approval of manuscript

Lu Chen: Conception and design, Data analysis and interpretation, manuscript writing, final approval of manuscript

Guiqiang Fu: Collection and/or assembly of data, data analysis and interpretation, manuscript writing, final approval of manuscript

Wenze Chen^:^ Collection and/or assembly of data, data analysis and interpretation, manuscript writing, final approval of manuscript

Zitong Yang: Collection and/or assembly of data, data analysis and interpretation, manuscript writing, final approval of manuscript

Wenjing Li: Collection and/or assembly of data, manuscript writing, final approval of manuscript

Yuqiong Zhou: Data analysis and interpretation, manuscript writing, final approval of manuscript

Jiacheng Jin: Data analysis and interpretation, manuscript writing, final approval of manuscript

Linxi Zhou: Conception and design, financial support, data analysis and interpretation, manuscript writing, final approval of manuscript

Duohong Zou: Conception and design, financial support, provision of study material or patients, data analysis and interpretation, manuscript writing, final approval of manuscript

All authors have read and approved the final manuscript.

Corresponding Authors:

Duohong Zou, MD, PhD, Department of Oral Surgery, Ninth People’s Hospital, Shanghai Jiao Tong University School of Medicine, 639 Zhizaoju Road, Shanghai, 200011, China.

Telephone number: (+86)13601645549

Email: zouduohongyy@163.com

Linxi Zhou, PhD, Department of Orthodontics, Shanghai Ninth People's Hospital, Shanghai Jiao Tong University School of Medicine, 639 Zhizaoju Road, Shanghai, 200011, China

Telephone number: (+86)15000370554

Email: [jolenee@sjtu.edu.cn](mailto:jolenee@sjtu.edu.cn)


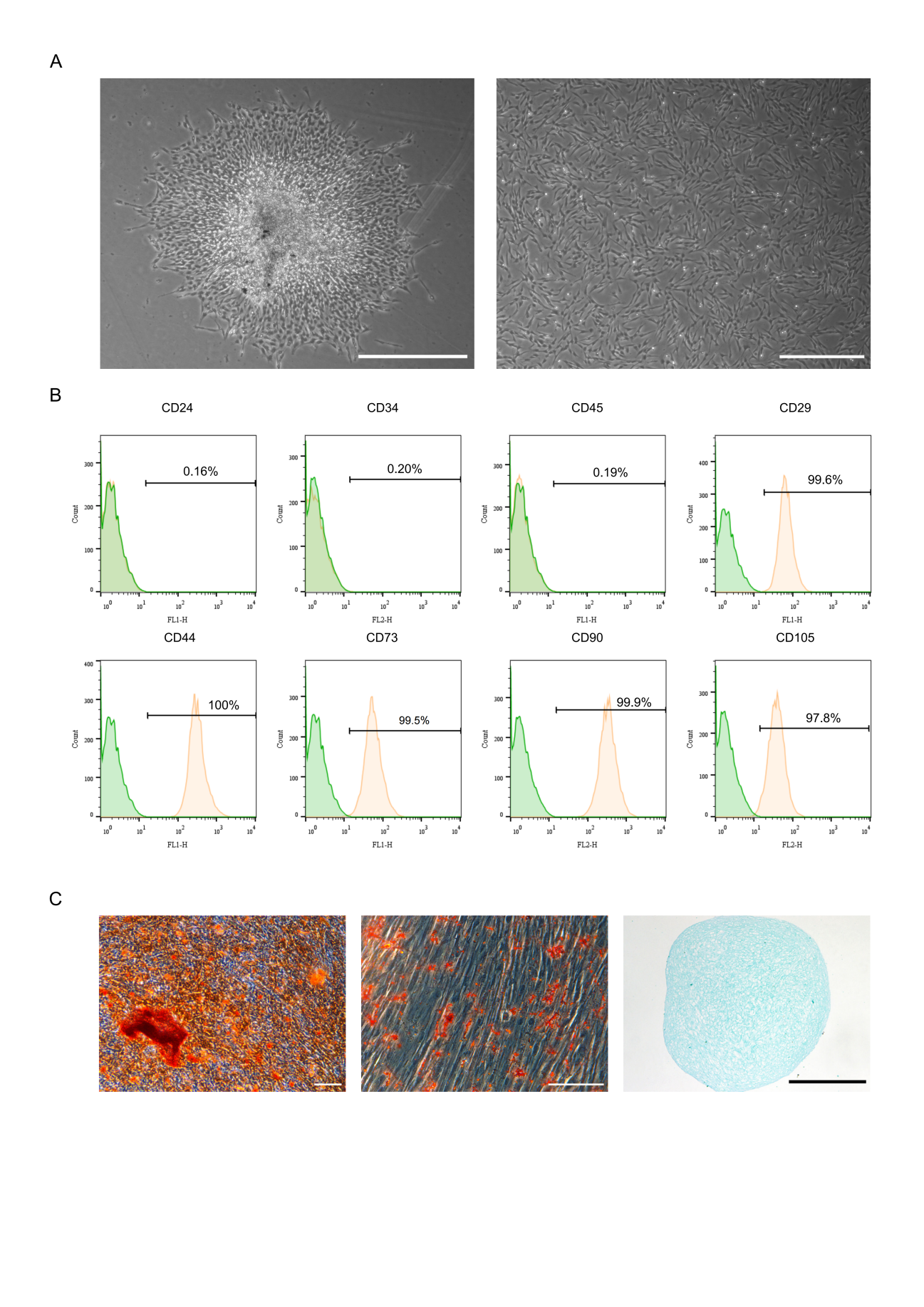


**Figure S1. Isolation and characterization of DPSCs**

1. Cell morphology of DPSCs in P0 and P3. The scale bars correspond to 500 μm. **(B)** Flow cytometric analysis of DPSCs expressed surface molecules. **(C)** Alizarin Red Staining of osteogenic differentiated DPSCs (the left). Oil Red O staining of lipogenic differentiated DPSCs (the middle). Alcian Blue staining of chondrogenic differentiated DPSCs (the right). The scale bars correspond to 100 μm. DPSCs, dental pulp stem cells; P0, passage 0; P3, passage 3.

**Alt text**: Characterization of dental pulp stem cells (DPSCs). A, Cell morphology at passage 0 and passage 3. B, Flow cytometric analysis of surface molecule expression. C, Alizarin Red staining of osteogenic differentiated DPSCs, Oil Red O staining of adipogenic differentiated DPSCs, and Alcian Blue staining of chondrogenic differentiated DPSCs.


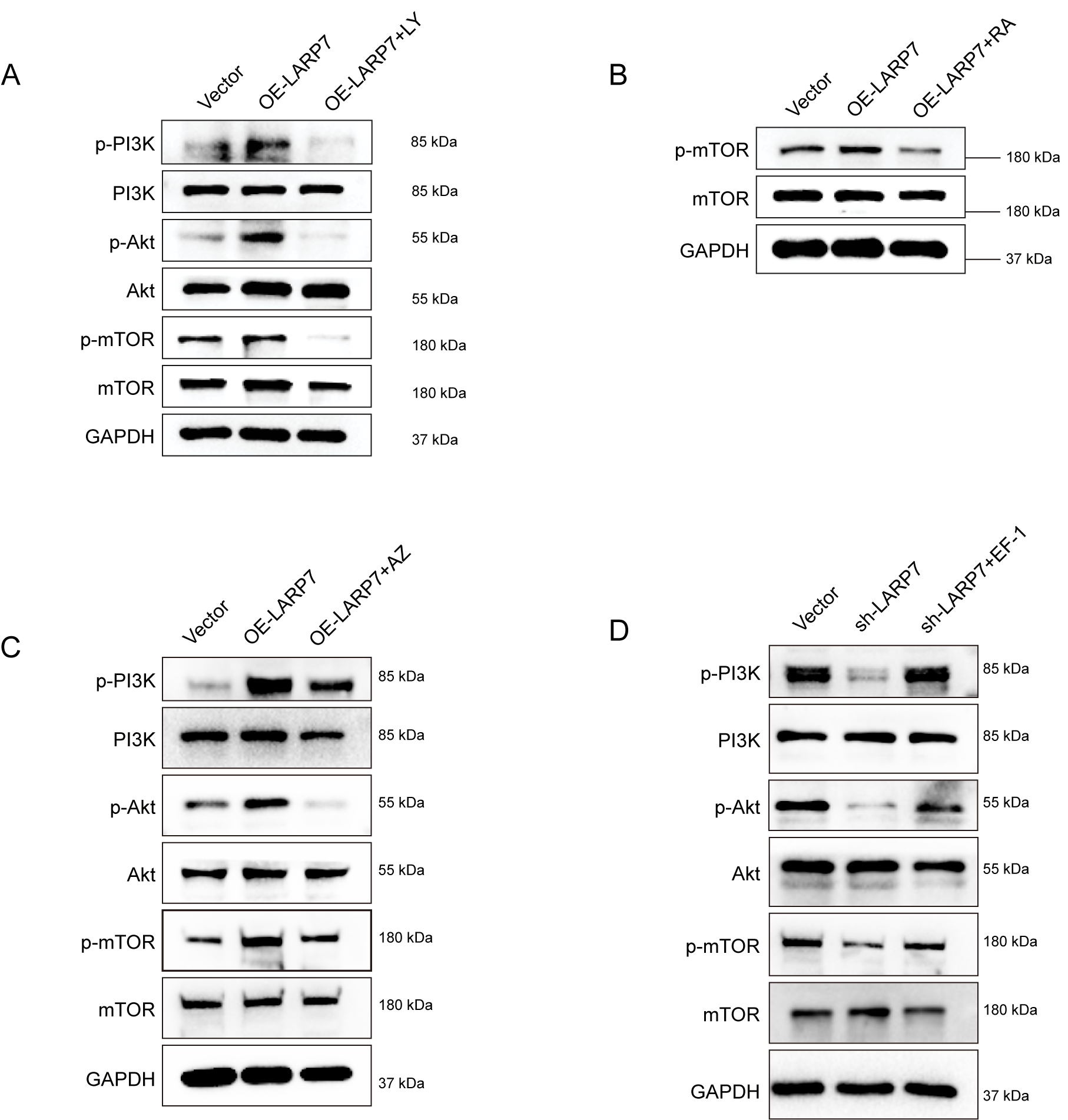


**Figure S2. LARP7 activated the PI3K-Akt-mTOR signaling**

Following lentiviral-mediated LARP7 knockdown or overexpression, DPSCs were subjected to neural induction for 3 days in the presence of pathway-specific inhibitors: LY294002 (25 μM, PI3K-Akt inhibitor), rapamycin (200 nM, mTOR inhibitor), AZ-5104 (10 nM, ERBB4 inhibitor), or EF-1 (10 μM, ERBB4 agonist). **(A)** The protein expression levels of p-PI3K, PI3K, p-Akt, Akt, p-mTOR and mTOR were evaluated by WB. **(B)** The protein expression levels of p-mTOR and mTOR were evaluated by WB. **(C, D)** The protein expression levels of p-PI3K, PI3K, p-Akt, Akt, p-mTOR and mTOR were evaluated by WB.

Full-length blots are presented in Figure S8. Bands for each protein represent results from parallel independent Western Blot experiments using aliquots of the same sample, each accompanied by its own GAPDH loading control lane.

**Alt text:** Validation of PI3K-Akt-mTOR pathway inhibition. A, Western blot shows p-PI3K, PI3K, p-Akt, Akt, p-mTOR, and mTOR expression with LY294002 treatment. B, Western blot shows p-mTOR and mTOR expression with rapamycin treatment.

**
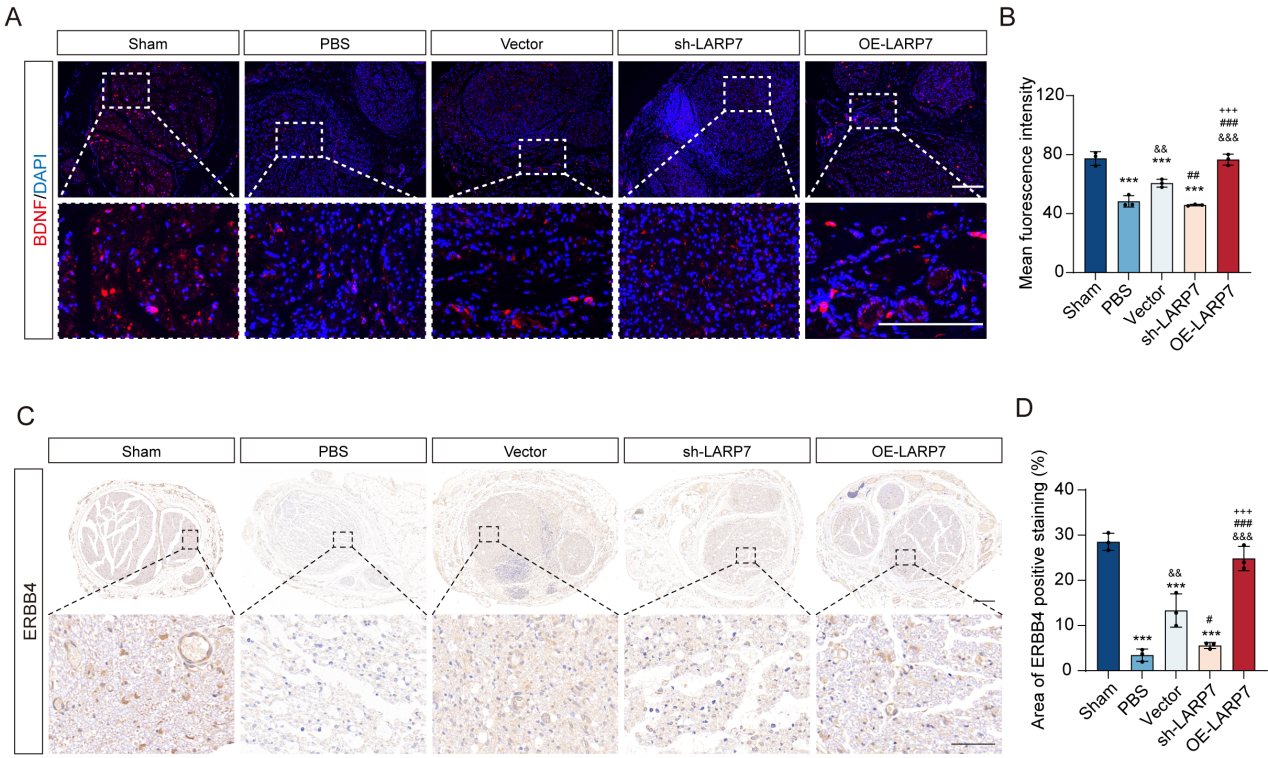
**

**Figure S3.** **LARP7-overexpressed DPSCs improved the repair of PNIs**

**(A)** Representative images show BDNF (red) fluorochrome labels in cross-section of sciatic nerves. Nuclei were stained with DAPI (blue). The scale bars correspond to 100 μm. **(B)** The expression levels of BDNF detected by Immunofluorescence were statistically quantified (n = 3). **(C)** ERBB4 expressed in the cross-sections of sciatic nerves are presented in the images. Scale bars, 50 μm. **(D)** The expression levels of ERBB4 detected by immunohistochemistry were statistically quantify (n = 3)

For all charts, ^*^, each group vs. Sham group. ^&^, each group vs. PBS group. ^#^, each group vs. Vector group. ^+^, each group vs. sh-LARP7 group. *^*^p*<0.05, *^**^p*<0.01, *^***^p*<0.001. ^&^*p*<0.05, ^&&^*p*<0.01, ^&&&^*p*<0.001. ^#^*p*<0.05, ^##^*p*<0.01, ^###^*p*<0.001. ^+^*p*<0.05, ^++^*p*<0.01, ^+++^*p*<0.001. Error bars indicate SD.

**Alt text:** LARP7-overexpressing DPSCs increase BDNF and ERBB4 expression in *vivo*. A, Immunofluorescence shows BDNF expression in sciatic nerve cross-sections from Sham, PBS, Vector, sh-LARP7, and OE-LARP7 groups. B, Quantification of BDNF fluorescence intensity in each group. C, Immunohistochemistry shows ERBB4 expression in sciatic nerve cross-sections from each group. D, Quantification of ERBB4 expression in each group.


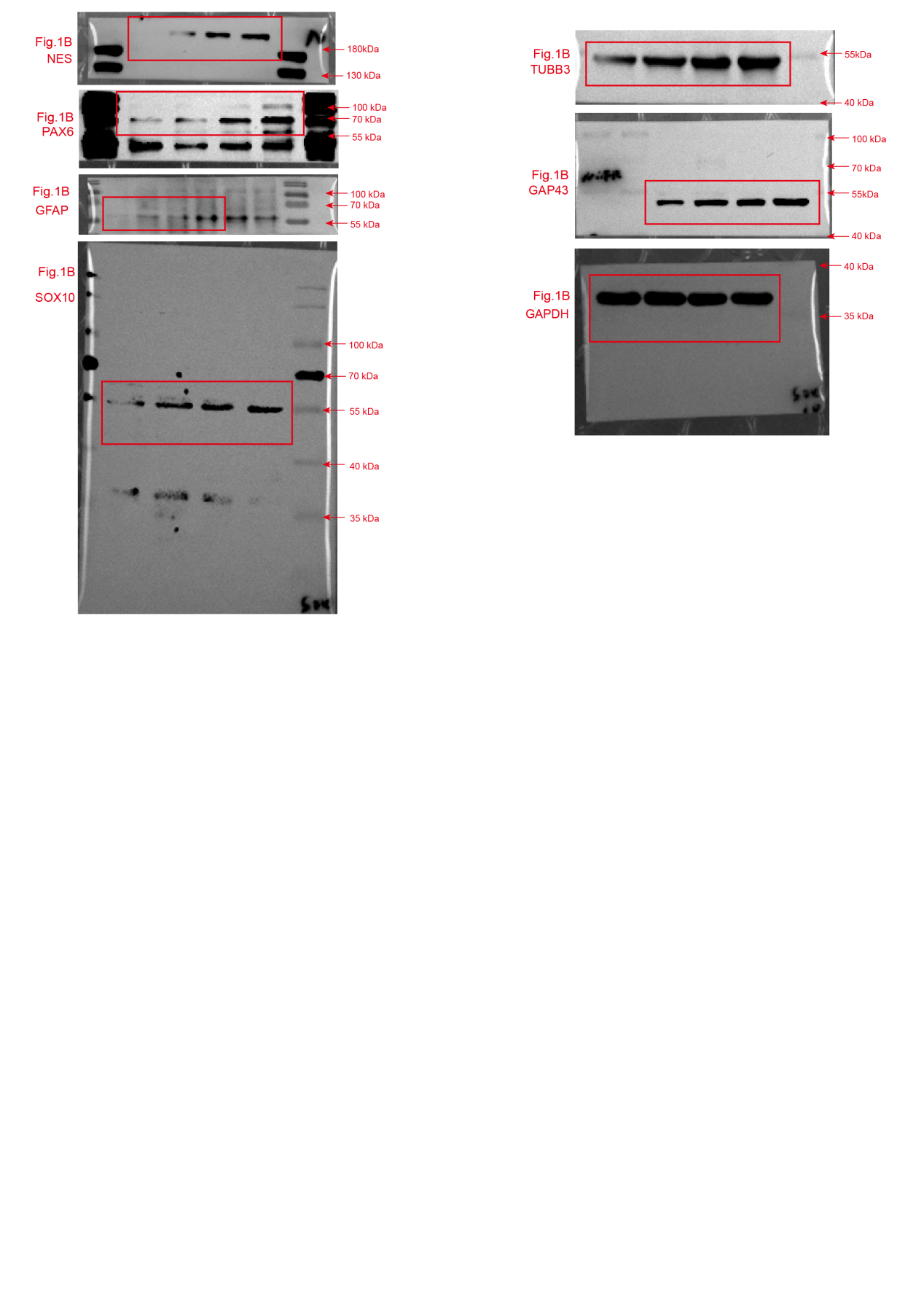


**Figure S4. Full-length blots of Figure 1**

**
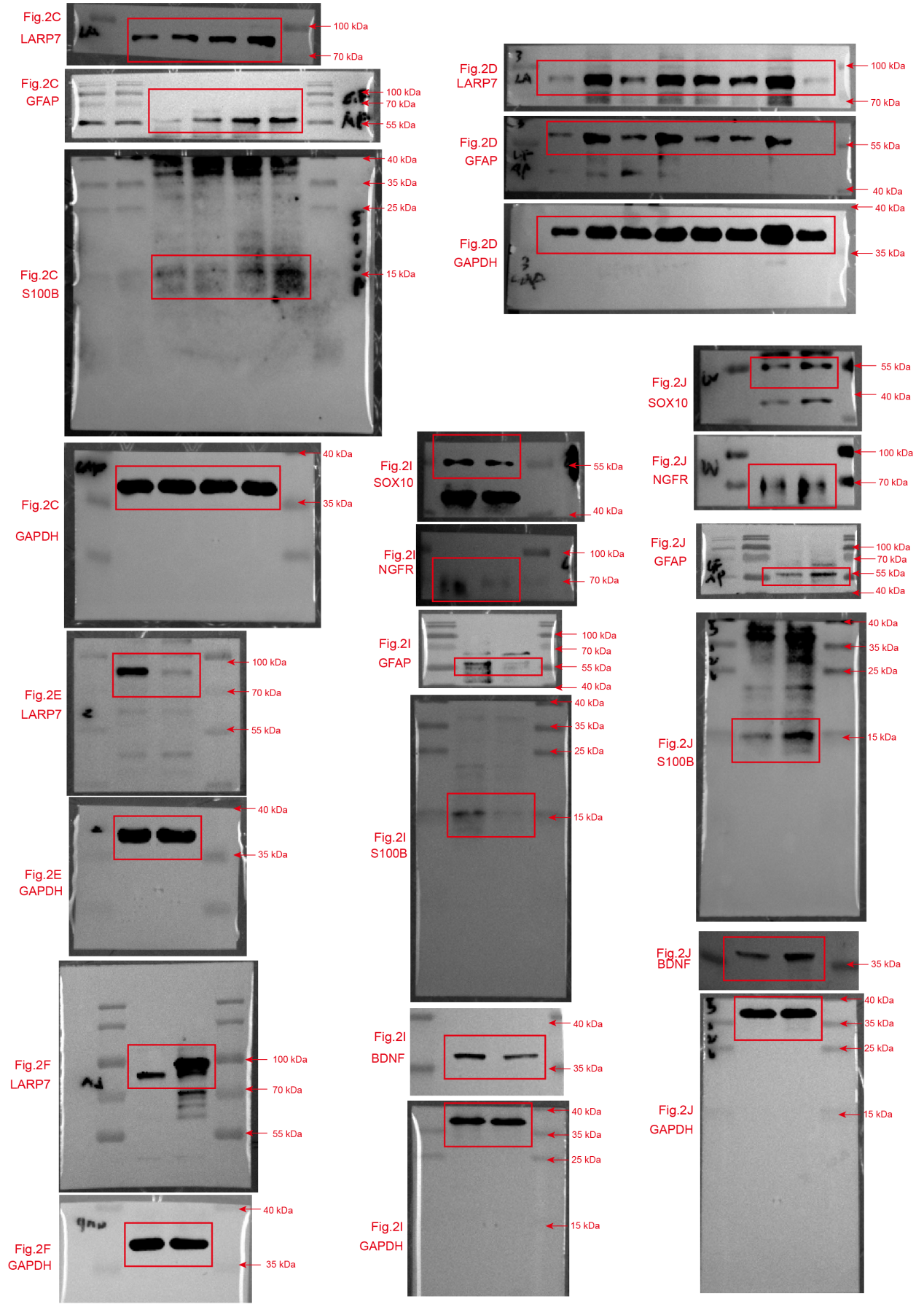
**

**Figure S5. Full-length blots of Figure 2**

**
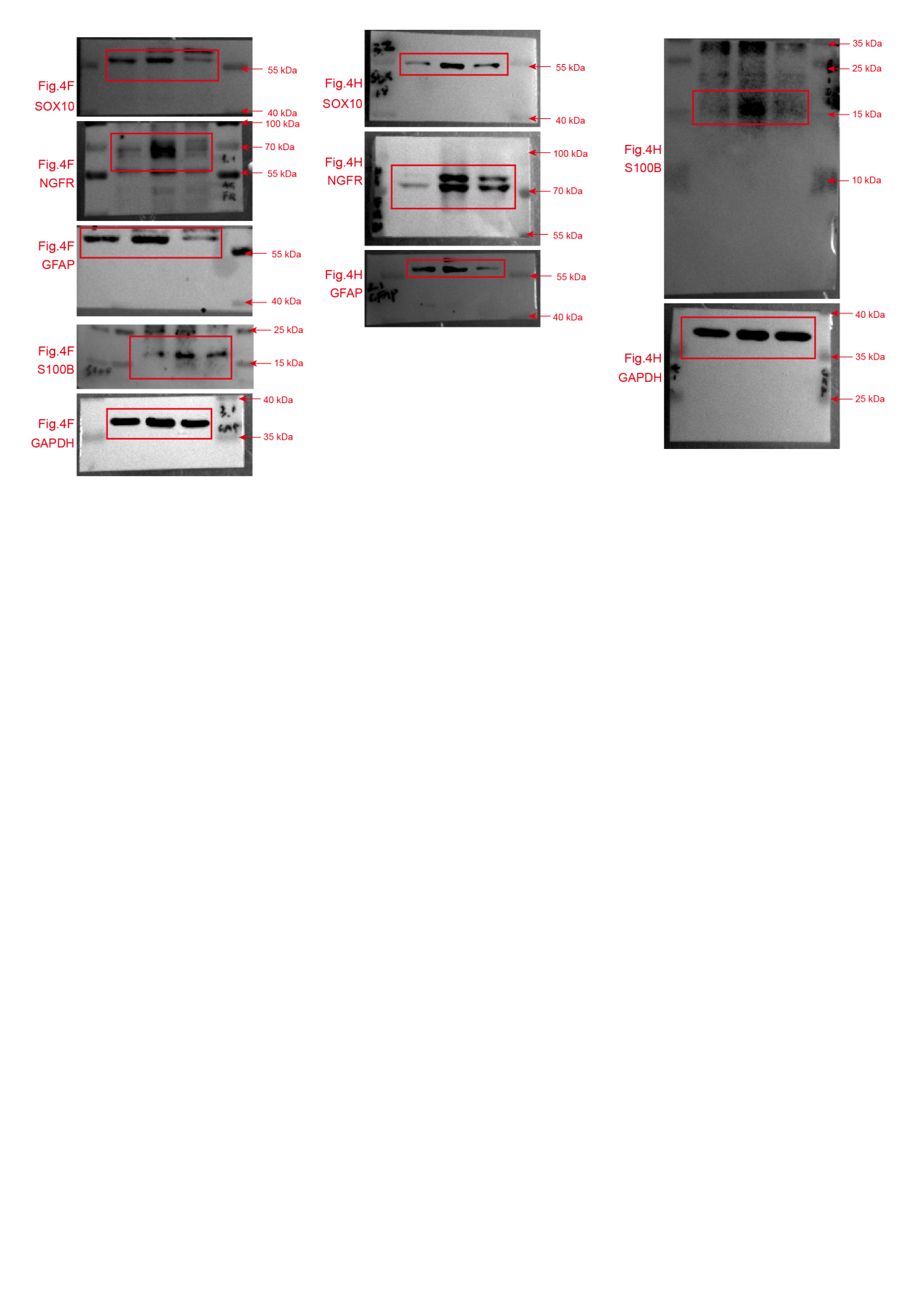
**

**Figure S6. Full-length blots of Figure 4**

**
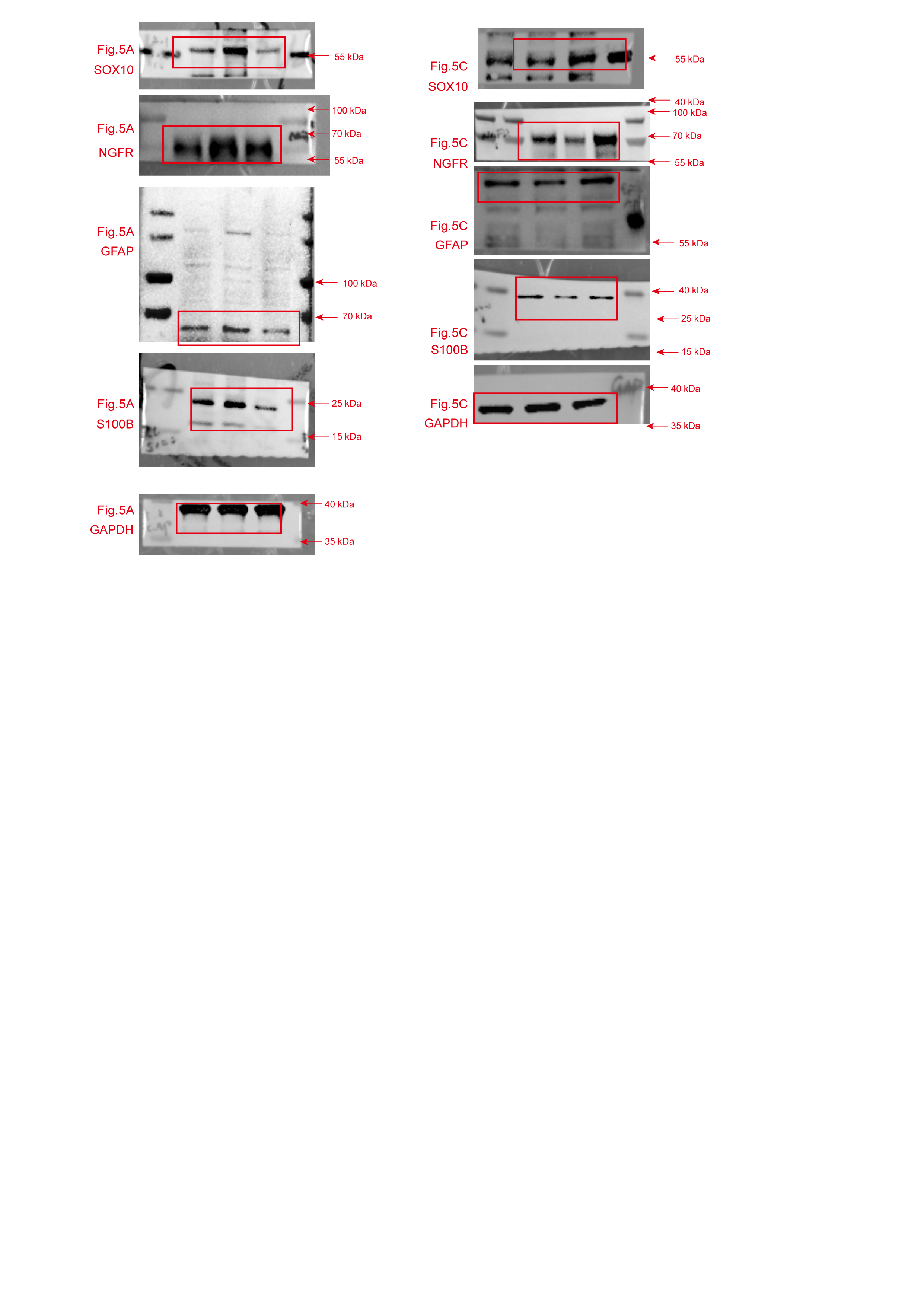
**

**Figure S7. Full-length blots of Figure 5**

**
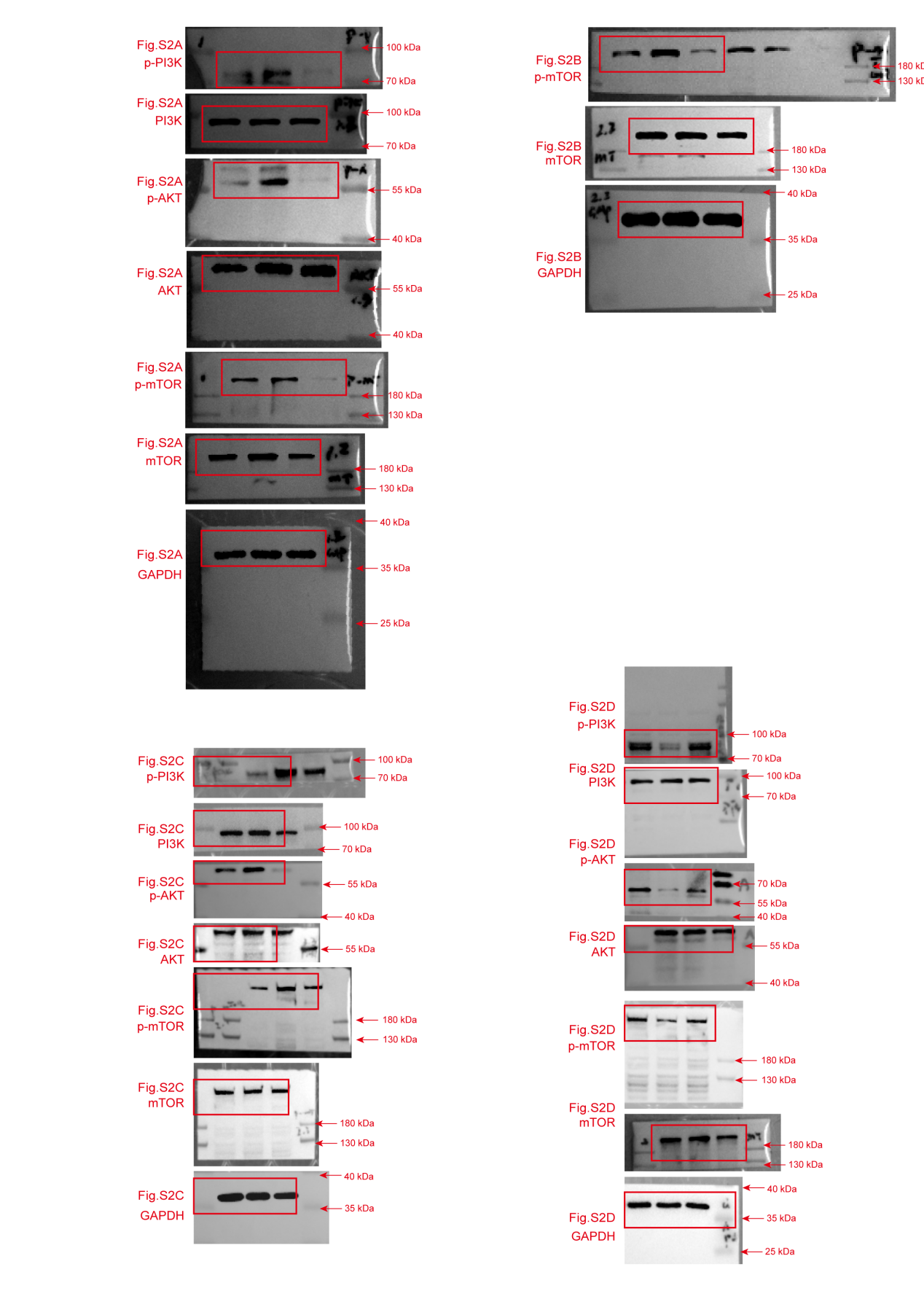
**

**Figure S8. Full-length blots of Figure S2**

| **Table S1 The primers used in the qRT-PCR assays** | | |
| --- | --- | --- |
| Gene | | Sequence |
| *EP300* | Forward primer | AGCCAAGCGGCCTAAACTC |
|  | Reverse primer | TCACCACCATTGGTTAGTCCC |
| *ETS1* | Forward primer | GATAGTTGTGATCGCCTCACC |
|  | Reverse primer | GTCCTCTGAGTCGAAGCTGTC |
| *PCGF1* | Forward primer | CGCTACGGAACGAGGAGGA |
|  | Reverse primer | CCGGTCCAGTTTGAGGTTGAG |
| *SP1* | Forward primer | TGGCAGCAGTACCAATGGC |
|  | Reverse primer | CCAGGTAGTCCTGTCAGAACTT |
| *SP2* | Forward primer | CTCAGCCCCGGCAAGAATAG |
|  | Reverse primer | TTGATCGGGTCCCTTTGTTGA |
| *TET2* | Forward primer | GATAGAACCAACCATGTTGAGGG |
|  | Reverse primer | TGGAGCTTTGTAGCCAGAGGT |
| *ANGPTL1* | Forward primer | AGTGGACACTGGACATTGCAG |
|  | Reverse primer | GCTTCCTCTTTACCATCTGTGG |
| *CCL2* | Forward primer | CAGCCAGATGCAATCAATGCC |
|  | Reverse primer | TGGAATCCTGAACCCACTTCT |
| *DEPDC1B* | Forward primer | AGACCGTGGAGCTTTTTCGTG |
|  | Reverse primer | TTCAGGGCCGAAGTTTTGACT |
| *GPRC5A* | Forward primer | ATGGCTACAACAGTCCCTGAT |
|  | Reverse primer | CCACCGTTTCTAGGACGATGC |
| *IDO1* | Forward primer | GCCAGCTTCGAGAAAGAGTTG |
|  | Reverse primer | ATCCCAGAACTAGACGTGCAA |
| *IL4L1* | Forward primer | GCCAAGACCCCTTCGAGAAAT |
|  | Reverse primer | CCGATCCTGTTATCTGCCTCC |
| *TCN2* | Forward primer | GGCCCTCACTGAGATGTGTG |
|  | Reverse primer | CTGTGCAGGTAGAGGTCTTCC |
| *TMEM100* | Forward primer | TGCTGTGGTTGTCTTCATCG |
|  | Reverse primer | CTCTCCCGTCTCTTGGCTTTC |
| *COL6A1* | Forward primer | ACAGTGACGAGGTGGAGATCA |
|  | Reverse primer | GATAGCGCAGTCGGTGTAGG |
| *CSF1* | Forward primer | TGGCGAGCAGGAGTATCAC |
|  | Reverse primer | AGGTCTCCATCTGACTGTCAAT |
| *ERBB4* | Forward primer | GTCCAGCCCAGCGATTCTC |
|  | Reverse primer | AGAGCCACTAACACGTAGCCT |
| *KDR* | Forward primer | GGCCCAATAATCAGAGTGGCA |
|  | Reverse primer | CCAGTGTCATTTCCGATCACTTT |
| *PGF* | Forward primer | GAACGGCTCGTCAGAGGTG |
|  | Reverse primer | ACAGTGCAGATTCTCATCGCC |
| *PDGFD* | Forward primer | TTGTACCGAAGAGATGAGACCA |
|  | Reverse primer | GCTGTATCCGTGTATTCTCCTGA |
| *BCOR* | Forward primer | TGGTGACGCTTCAAAAGCCA |
|  | Reverse primer | GCTAGAATAGACGATGTTTCCCG |
| *BRD4* | Forward primer | ACCTCCAACCCTAACAAGCC |
|  | Reverse primer | TTTCCATAGTGTCTTGAGCACC |
| *CTCF* | Forward primer | CAGTGGAGAATTGGTTCGGCA |
|  | Reverse primer | CTGGCGTAATCGCACATGGA |
| *EHMT2* | Forward primer | GGGCGGGAAAATCACCTCC |
|  | Reverse primer | CACTCATGCGGAAATGCTGTAT |
| *LARP7* | Forward primer | CGGTCACGAGTTAAACAGGTG |
|  | Reverse primer | GCCTTCCAAATCAAGCTCTACAA |
| *GFAP* | Forward primer | CTGCGGCTCGATCAACTCA |
|  | Reverse primer | TCCAGCGACTCAATCTTCCTC |
| *SOX10* | Forward primer | CCTCACAGATCGCCTACACC |
|  | Reverse primer | CATATAGGAGAAGGCCGAGTAGA |
| *S100B* | Forward primer | TCTGGAAGGGAGGGAGACAA |
|  | Reverse primer | GGAAGTCACATTCGCCGTCT |
| *NGFR* | Forward primer | CCGTTGGATTACACGGTCCAC |
|  | Reverse primer | TGAAGGCTATGTAGGCCACAA |
| *BDNF* | Forward primer | GGCTTGACATCATTGGCTGAC |
|  | Reverse primer | CATTGGGCCGAACTTTCTGGT |
| *GDNF* | Forward primer | GGCAGTGCTTCCTAGAAGAGA |
|  | Reverse primer | AAGACACAACCCCGGTTTTTG |
| *VEGFA* | Forward primer | AGGGCAGAATCATCACGAAGT |
|  | Reverse primer | AGGGTCTCGATTGGATGGCA |
| *IL-10* | Forward primer | GACTTTAAGGGTTACCTGGGTTG |
|  | Reverse primer | TCACATGCGCCTTGATGTCTG |
| *IL-6* | Forward primer | ACTCACCTCTTCAGAACGAATTG |
|  | Reverse primer | CCATCTTTGGAAGGTTCAGGTTG |
| *FIL1* | Forward primer | TTTGCCTGAAATGGTGAGTAAGG |
|  | Reverse primer | TGGTTTGCTTGAGCTGTGTTC |
| *IL-10RA* | Forward primer | CCTCCGTCTGTGTGGTTTGAA |
|  | Reverse primer | CACTGCGGTAAGGTCATAGGA |
| *IL-6R* | Forward primer | CCCCTCAGCAATGTTGTTTGT |
|  | Reverse primer | CTCCGGGACTGCTAACTGG |
| *ACTB* | Forward primer | TGGCACCACACCTTCTACAATGAGC |
|  | Reverse primer | GCACAGCTTCTCCTTAATGTCACGC |
